# ParticleChromo3D: a Particle Swarm Optimization algorithm for chromosome 3D structure prediction from Hi-C data

**DOI:** 10.1186/s13040-022-00305-x

**Published:** 2022-09-21

**Authors:** David Vadnais, Michael Middleton, Oluwatosin Oluwadare

**Affiliations:** Department of Computer Science, University of Colorado, Colorado Springs, CO USA

**Keywords:** Hi-C, 3D chromosome structure, Particle Swarm Optimization, Chromosome conformation capture, 3D genome

## Abstract

**Background:**

The three-dimensional (3D) structure of chromatin has a massive effect on its function. Because of this, it is desirable to have an understanding of the 3D structural organization of chromatin. To gain greater insight into the spatial organization of chromosomes and genomes and the functions they perform, chromosome conformation capture (3C) techniques, particularly Hi-C, have been developed. The Hi-C technology is widely used and well-known because of its ability to profile interactions for all read pairs in an entire genome. The advent of Hi-C has greatly expanded our understanding of the 3D genome, genome folding, gene regulation and has enabled the development of many 3D chromosome structure reconstruction methods.

**Results:**

Here, we propose a novel approach for 3D chromosome and genome structure reconstruction from Hi-C data using Particle Swarm Optimization (PSO) approach called ParticleChromo3D. This algorithm begins with a grouping of candidate solution locations for each chromosome bin, according to the particle swarm algorithm, and then iterates its position towards a global best candidate solution. While moving towards the optimal global solution, each candidate solution or particle uses its own local best information and a randomizer to choose its path. Using several metrics to validate our results, we show that ParticleChromo3D produces a robust and rigorous representation of the 3D structure for input Hi-C data. We evaluated our algorithm on simulated and real Hi-C data in this work. Our results show that ParticleChromo3D is more accurate than most of the existing algorithms for 3D structure reconstruction.

**Conclusions:**

Our results also show that constructed ParticleChromo3D structures are very consistent, hence indicating that it will always arrive at the global solution at every iteration. The source code for ParticleChromo3D, the simulated and real Hi-C datasets, and the models generated for these datasets are available here: https://github.com/OluwadareLab/ParticleChromo3D

**Supplementary Information:**

The online version contains supplementary material available at 10.1186/s13040-022-00305-x.

## Background

Understanding the three-dimensional (3D) architecture of the genome is essential for understanding a variety of biological processes such as gene expression, gene stability and regulation, and DNA replication [1, 2]. To aid the genome architecture study, Chromosome Conformation Capture (3C) and its derivative technologies have been extremely beneficial in defining the 3D structure of the genome [1]. Because of its systematic nature, 3C's biochemical technique to investigating DNA's topography within chromatin has outperformed traditional microscopy tools such as fluorescence in situ hybridization (FISH) in aiding the 3D genome structure study [2]. It is worth noting that microscopy approaches are sometimes used in conjunction with 3C for verifying the 3D structure of a chromosome or genome [1]. 3C was first described by [3] Dekker et al. (2002). Since then, more technologies were developed [4], such as the Chromosome Conformation Capture-on-Chip (4C) [5], Chromosome Conformation Capture Carbon Copy (5C) [6], Hi-C [7], Tethered Conformation Capture (TCC) [8], and Chromatin Interaction Analysis by Paired-End Tag sequencing (ChIA-PET) [2, 9]. These derivative technologies were designed to augment 3C's in the following areas, measure spatial data within chromatin, increase measuring throughput, and analyze proteins and RNA within chromatin instead of just DNA. Lieberman-Aiden et al., 2009 [7] designed high-throughput chromosome conformation capture technology (Hi-C) as a minimally biased "all vs. all" approach. Hi-C works by injecting biotin-labeled nucleotides during the ligation step [4]. Hi-C provides a method for finding genome-wide chromatin Information Frequency (IF) data in the form of a contact matrix [1]. In addition, the Hi-C datasets provides important information for visualizing regulatory element interactions at specific loci or depicting the hierarchical organization of nuclear genome structure, which are more observable in a 3D structure.

Hi-C analysis doubtlessly introduced great benefit to 3D genome research— they explain a series of events such as genome folding, gene regulation, genome stability, and the relationship between regulatory elements and structural features in the cell nucleus [2, 7, 10]. Importantly, it is possible to glean insight into chromatin's 3D structure using the Hi-C data. However, to use Hi-C data for 3D structure modeling, some pre-processing is necessary to extract the interaction frequencies between the chromosome or genome’s interacting loci [11]. This process involves quality control and mapping of the data [12]. Once these steps are completed, an IF matrix, or called contact matrix or map, is generated. An IF matrix is a symmetric matrix that records a one-to-one interaction frequency for all the intersecting loci [7, 10]. The IF matrix is represented as either a square contact matrix or as a three-column sparse matrix. Each cell has genomic bins within these matrices that are the length of the data's resolution representing each cell [12]. Hence, the higher the resolution (5 KB), the larger the contact matrix's size. And similarly, the lower the resolution (1 MB), the smaller the contact matrix's size. Next, this Hi-C data is normalized to remove biases that next-generation sequencing can create [12, 13]. An example of this type of bias would be copy number variation [13]. Other systematic biases introduced during the Hi-C experiment are external factors, such as DNA shearing and cutting [10]. Today, several computational algorithms have been developed to remove these biases from the Hi-C IF data [13–19]. Once the Hi-C IF matrix data is normalized, it is most suitable for 3D chromosome or genome modeling. Some tools have been developed to automate these Hi-C pre-processing steps; they include GenomeFlow [20], Hi-Cpipe [21], Juicer [22], HiC-Pro [23], and HiCUP [24].

To create 3D chromosome and genome structures from IF data, many techniques can be used. Oluwadare, O., et al*.* (2019) [10] pooled the various developed analysis techniques into three buckets, which are Distance-based, Contact-based, and Probability-based methods. The first method is a Distance-based method that maps IF data to distance data and then uses an optimizer to solve for the 3D coordinates [12]. This type of analysis's final output will be (x, y, z) coordinates [12]. An advantage of Distance-based methods is that they are unambiguous in their results and because of this the relative accuracy of algorithms can be easily compared [10]. This unambiguity helps make distance-based algorithms useful for managing a large range of modeling problems with different resolutions and noise thresholds [10]. However, the difficulty is picking out how to convert the IF data and which optimization algorithm to use [10]. The distance between two genomic bins is often represented as ${Distance}_{i,j}$= 1/$({{IF}_{i,j}}^{\alpha })$ [10, 11]. In this approach ${IF}_{i,j}$ is the number of times two genomic bins had contact, and $\alpha$ is a factor which is used for modeling, called the conversion factor. This distance can then be optimized against other genomic bins' other distance values to create a 3D model. Several methods [10] belong in this category include, ChromSDE [25], AutoChrom3D [26], Chromosome3D [27], 3DMax [28], ShRec3D [29], LorDG [30], InfMod3DGen [31], HSA [32], ShNeigh [33]. The second classification for 3D genome structure modeling algorithms from IF data is Contact-based methods. This technique uses the IF data directly instead of starting by converting the data to an (x, y, z) coordinate system [10]. One way to model this data is with a gradient descent/ascent algorithm [10]. This approach was explored by Trieu T, and Cheng J., 2015 through the algorithm titled MOGEN [34]. MOGEN works by optimizing a scoring function that scores how well the chromosomal contact rules have been satisfied [34]. This method has a strength in its scalability [10, 34]. Scalability is especially important for ensemble models [10]. Another contact method was to take the interaction frequency and use it for spatial restraints [35]. Gen3D [36], Chrom3D [37], and GEM [38] are other examples in this category. The third classification is Probability-based. The advantages of probability-based approaches are that they easily account for uncertainties in experimental data and can perform statistical calculations of noise sources or specific structural properties [10]. Unfortunately, probability techniques can be very time-consuming compared to Contact and Distance methods. Rousseau et al., 2011 created the first model in this category using a Markov chain Monte Carlo approach called MCMC5C [39]. Markov chain Monte Carlo was used due to its synergy with estimating properties' distribution [10]. Varoquaux. N. et al*.,* 2014 [40] extended this probability-based approach to modeling the 3D structure of DNA. They used a Poisson model and maximized a log-likelihood function [40]. Many other statistical models can still be explored.

This paper presents ParticleChromo3D, a new distance-based algorithm for chromosome 3D structure reconstruction from Hi-C data. ParticleChromo3D uses Particle Swarm Optimization (PSO) to generate 3D structures of chromosomes from Hi-C data. We chose PSO because of its social and individual qualities, which we hypothesis will allow it to optimize sub-sections of the chromosome while conveying its progress to the entire swarm. Given the correct topology and coefficient optimization, this advantage could help advance the state of the art for avoiding local minima. The sub-section “*Why PSO”* of the *Methods section* delves deeper into the rationale for using the PSO algorithm and the gap it addresses, especially for distance-based optimization approaches that focus on global optimizations. Here, we show that ParticleChromo3D can generate candidate structures for chromosomes from Hi-C data. Additionally, we analyze the effects of parameters such as confidence coefficient and swarm size (SS) on the structural accuracy of our algorithm. Finally, we compared ParticleChromo3D to a set of commonly used chromosome 3D reconstruction methods, and it performed better than most of these methods. We showed that ParticleChromo3D effectively generates 3Dstructures from Hi-C data and is highly consistent in its modeling performance.

## Methods

### The Particle Swarm Optimization algorithm

Kennedy J. and Eberhart R. (1995) [41] developed the Particle Swarm Optimization (PSO) as an algorithm that attempts to solve optimization problems by mimicking the behavior of a flock of birds. PSO has been used in the following fields: antennas, biomedical, city design/civil engineering, communication networks, combinatorial optimization, control, intrusion detection/cybersecurity, distribution networks, electronics and electromagnetics, engines and motors, entertainment, diagnosis of faults, the financial industry, fuzzy logic, computer graphics/visualization, metallurgy, neural networks, prediction and forecasting, power plants, robotics, scheduling, security and military, sensor networks, and signal processing [42–46]. Since PSO has been used in so many disparate fields, it appears robust and flexible, which gives credence to the idea that it could be used in this use case of bioinformatics and many others [47]. PSO falls into the optimization taxonomy of swarm intelligence [48]. PSO works by creating a set of particles or actors that explore a topology and look for the global minimum of that topology [48]. At each iteration, the swarm stores each particle's minimum result, as well as the global swarm's minimum, found. The particles explore the space with both a position and velocity, and they change their velocity based on three parameters. These three parameters are current velocity, distance to the personal best, and distance to the global best [48]. Position changes are made based on the calculated velocity during each iteration. The velocity function is as follows [49] in Eqs. (1) and (2):

1 $${V}_{n+1}=w*{V}_{n}+ {c}_{1}* {R}_{1}*\left( {P}_{n}- {X}_{n}\right)+ {c}_{2}* {R}_{2}*\left( {G}_{n}- {X}_{n}\right)$$

Then position is updated as follow:

2 $${X}_{n+1}= {X}_{n}+ {V}_{n+1}$$

where:

- ${V}_{n}$ is the current velocity at iteration $n$
- ${c}_{1}$ and ${c}_{2}$ are two real numbers that stand for local and global weights and are the personal best of the specific particle and the global best vectors, respectively, at iteration $n$ [49].
- The ${R}_{1}$ and ${R}_{2}$ values are randomized values used to increase the explored terrain [49].
- $w$ is the inertia weight parameter, and it determines the rate of contribution of a velocity [41].
- ${G}_{n}$ represents the best position of the swarm at iteration $n$.
- ${P}_{n}$ represents the best position of an individual particle.
- ${X}_{n}$ is the best position of an individual particle at the iteration $n$.

### Why PSO

This project's rationale is that using PSO could be a very efficient method for optimizing Hi-C data due to its inherent ability to hold local minima within its particles. This inherent property will allow sub-structures to be analyzed for optimality independently of the entire structure.

**Fig. 1:**
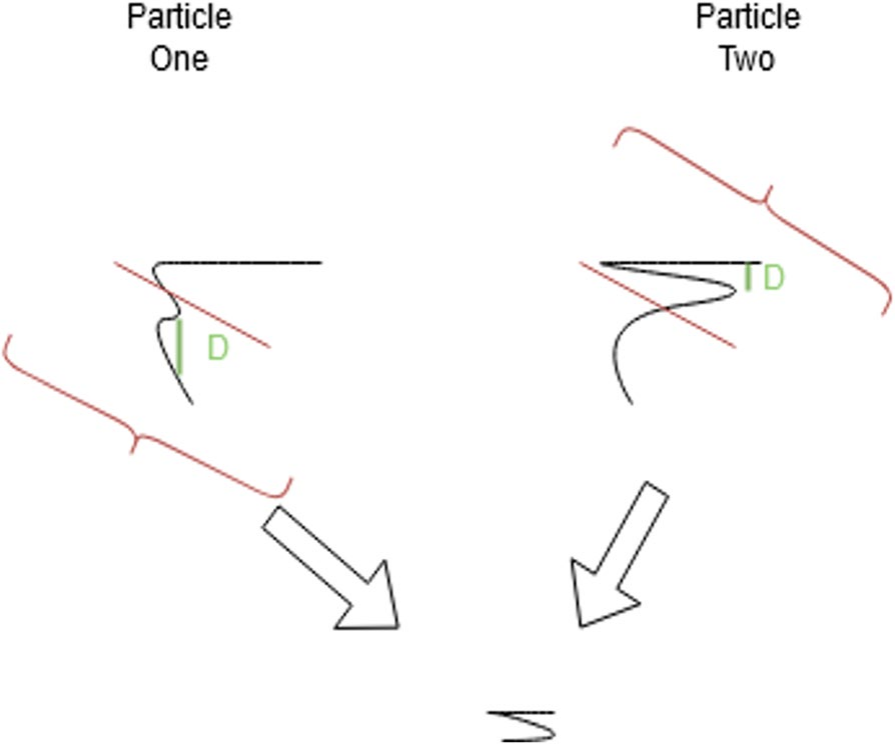
PSO potential advantage for structure folding. The figure summarizes the PSO algorithm performance expectation on the 3D genome structure reconstruction problem. The figure shows two particles with their current local 3D structure representations using example 3D structures. In this illustration, Particle one has reached the best global minimum, however Particle two has a better upper half that is theoretically independent of the bottom half. The figure depicts the 3D structure that will be created once the PSO algorithm traverses particle two towards the structure in particle one in the next iteration

**Fig. 2:**
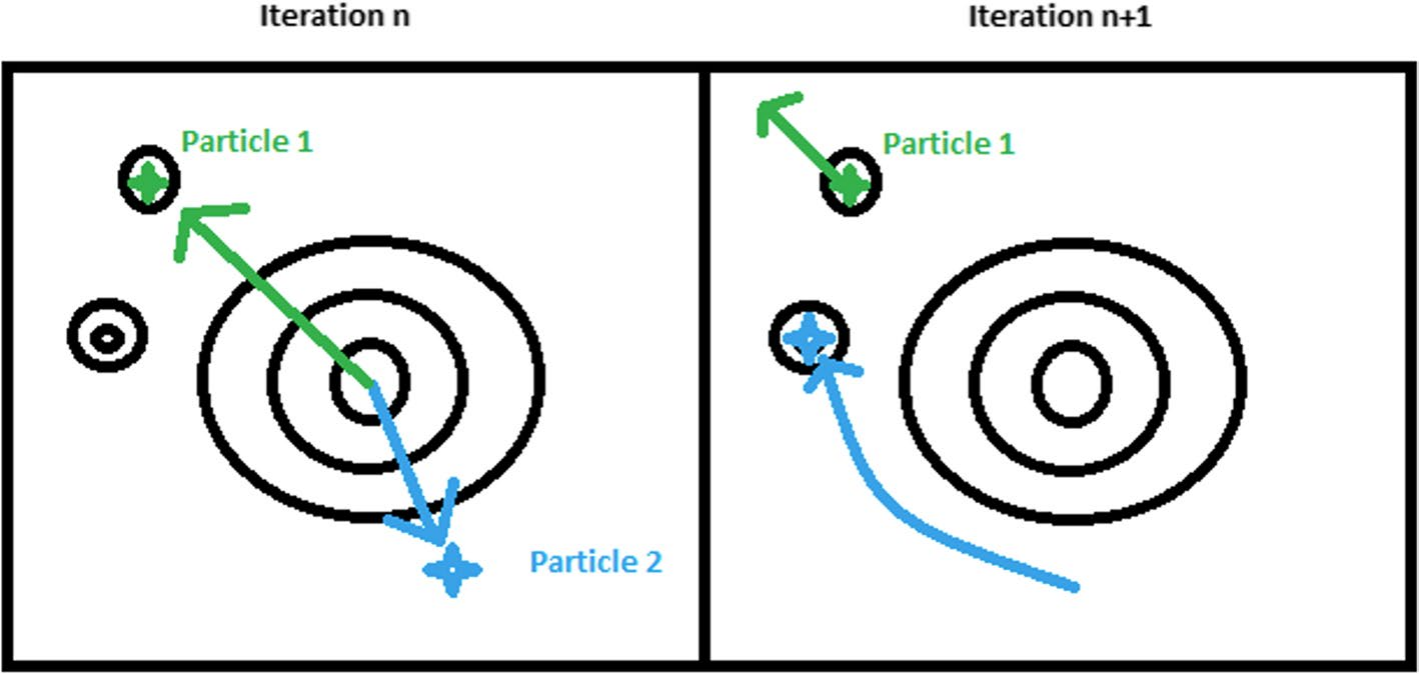
PSO particle iteration description. This figure explains the PSO algorithm's search mechanism for determining the best 3D structure following the individual particles' modified velocity and position in the swarm

In Fig. 1, particle one is at the global best minimum found so far. However, particle two has a better structure in its top half, and it is potentially independent of the bottom half. Because particle one has a better solution so far, particle two will traverse towards the structure in particle one in the iteration $n+1$. While particle two is traversing, it will go along a path that maintains its superior 3D model sections. Thus, it has a higher chance of finding the absolute minimum distance value. The more particles there are, the greater the time complexity of PSO and the higher the chance of finding the absolute minimum. The inherent breaking up of the problem could lend itself to powerful 3D structure creation results. More abstractly relative to Hi-C data but in the traditional PSO sense, the same problem as above might look as follows (Fig. 2) when presented in a topological map.

From Fig. 2, in the $nth$ iteration, particle 1 found a local minimum within this step. Since of all the particles, this is the lowest point; particle two will search towards particle one with a random chance amount added to its velocity [48, 50, 51]. The random chance keeps particle two from going straight to the optimal solution [48]. In this case, particle two found the absolute minima, and from here on, all the particles will begin to migrate towards particle 2. We will test this hypothesis by analyzing its output with the evaluation metrics defined in the “Methods” section.

In summary, we believe the particle-based structure of PSO may lend itself well to the problem of converting Hi-C IF data into 3D models. We will test this hypothesis and compare our results to the existing modeling methods.

### PSO for 3D structure reconstruction from Hi-C data

Here we describe how we implemented the PSO algorithm as a distance-based approach for 3D genome reconstruction from Hi-C data. This algorithm is called ParticleChromo3D. In this context, the input IF data is converted to the distance equivalent using the conversion factor, $\alpha$, for 3D structure reconstruction. These distances are sometimes called the "wish" distances [30] as they are a computational approximation of the 3D spatial distance between the underlying loci or bins in the chromosome. Because they are inferred from the input IF distance, they are used as the true representation to evaluate our algorithm's performance. That is, the closer our algorithm can predict these distances, the better it is. First, we initialize the particles' 3D (x,y,z) coordinates for each genomic bin or region randomly in the range [-1, 1]. Next, we set the stop parameters for our algorithm; these parameters are the maximum number of iterations allowed and the error threshold. For each bin, we now calculate a velocity and then update our position. We used the sum of squared error function as the loss function to compute chromosome structures from a contact map for the evaluation performed in this work. We described the impact of using different loss functions in the “Discussion” section.

**Fig. 3:**
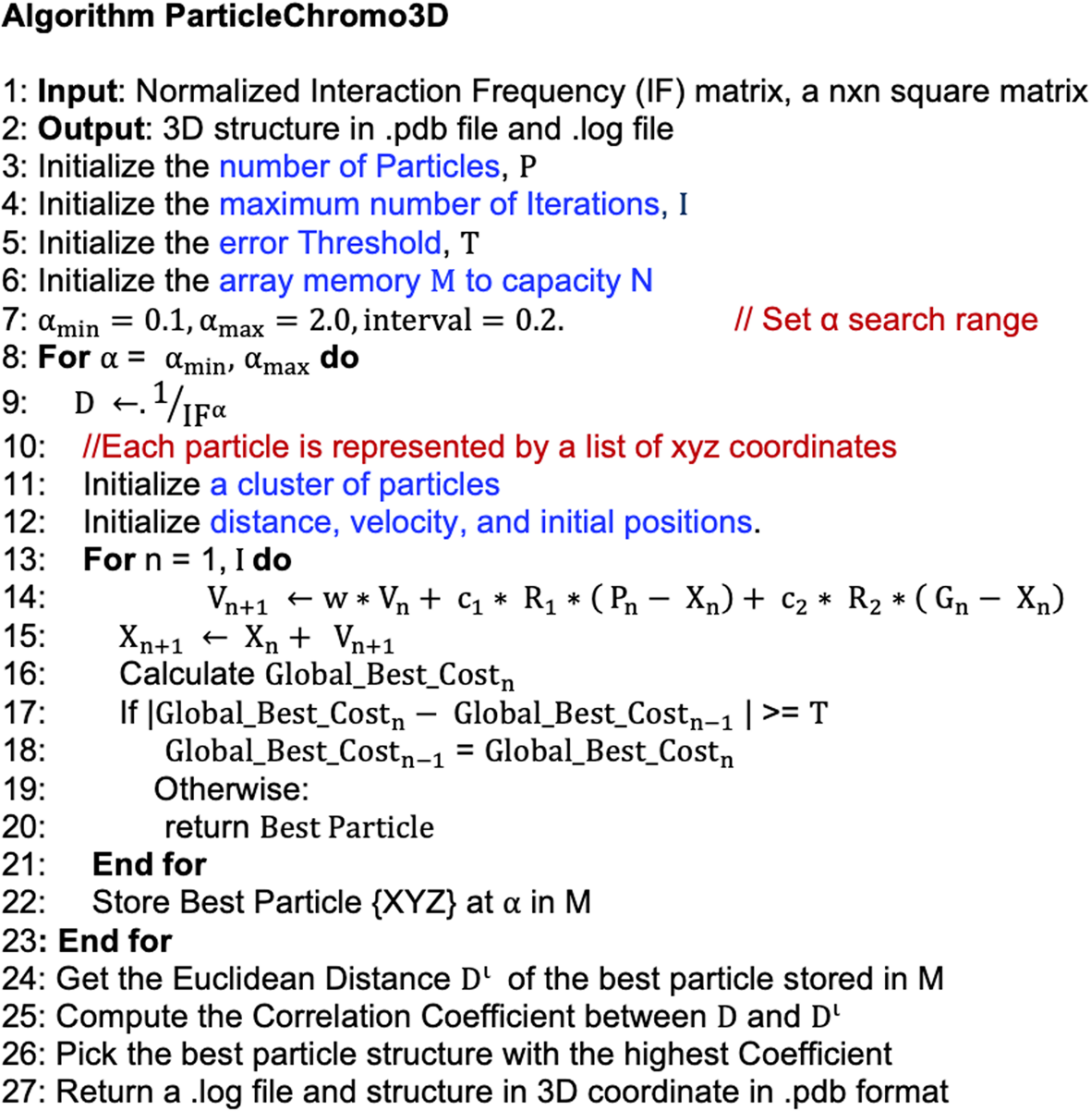
PSO for chromosome and genome 3D Structure prediction. We present a step-by-step illustration of the significant steps taken by ParticleChromo3D for 3D chromosome and genome structure reconstruction from an input normalized IF matrix

Finally, following the description provided for the PSO algorithm above, we used the PSO to iteratively improve our function until it has converged on either an absolute or local minimum. The complete ParticleChromo3D algorithm is presented in Fig. 3. Some parameters are needed to use the PSO algorithm for 3D structure reconstruction. This work has provided the parameter values that produced our algorithm's optimal results. The users can also provide their settings to fit their data where necessary. The results of the series of tests and validation performed to determine the default parameters are described in the "Parameters Estimation" section of the Methods section.

### Particle representation

A particle is a candidate solution. A list of XYZ coordinates represents each particle in the solution. The candidate solution's length in the number of regions in the input Hi-C data. Each particle's point is the individual coordinate, XYZ, of each bead. A swarm consists of N candidate solution, also called the swarm size, which the user provides as program input. We provide more explanation in the "Parameters Estimation" section below for how to determine the swarm size.

### Metrics used for evaluation

To evaluate the structure’s consistency with the input Hi-C matrix, we used the metrics below. All these metrics are represented in terms of distance. The evaluations were performed on the pairwise Euclidean distances between each chromosome locus corresponding to the structure generated by ParticleChromo3D and the pairwise distance of the underlying chromosome generated from the input Hi-C data.

### Distance Pearson Correlation Coefficient (DPCC)

The Distance Pearson correlation coefficient is as follows [10],

$$PCC=\frac{\sum (\left({d}_{i}-\overline{d }\right)*\left({D}_{i}-\overline{D }\right))}{\sqrt{\sum {\left({d}_{i}-\overline{d }\right)}^{2}* \sum {\left({D}_{i}-\overline{D }\right)}^{2}}}$$

where:

- ${D}_{i}$ and ${d}_{i}$ are instances of a distance value between two bins.
- $\overline{D}$ and $\overline{d }$ are the means of the distances within the data set.
- It measures the relationship between variables. Values a between -1 to + 1
- A higher value is better.

### Distance Spearman Correlation Coefficient (DSCC)

The Distance Spearman’s correlation coefficient is defined below [10],

$$SCC= \frac{\sum \left(x{}_{i}-\overline{x }\right)*({y}_{i}-\overline{y })}{\sqrt{\sum {\left({x}_{i}-\overline{x }\right)}^{2}}*\sqrt{\sum {\left({y}_{i}-\overline{y }\right)}^{2}}}$$

where:

- x_i_ and y_i_ are the rank of the distances,${D}_{i}$ and ${d}_{i},$ described in the DPCC equation above.
- $\overline{x }$ and $\overline{y }$ are the sample mean tank of both x and y, respectively.
- Values a between -1 to + 1. A higher value is better.

### Distance Root Mean Squared Error (DRMSE)

The Distance Root mean squared error follows the equation below [10],

$$RMSE= \sqrt{\frac{1}{n}*\sum {({d}_{i}-{D}_{i})}^{2}}$$

where:

- *D*_*i*_ and *d*_*i*_ are instances of distance values from the data and another data source.
- The value n is the size of the data set.

### TM-Score

TM-Score is defined as follows [52, 53],

$$TM-score= MAXIMUM\left[\frac{1}{{L}_{Target}}*{\sum }_{i}^{{L}_{ali}}\frac{1}{1+{(\frac{{\mathrm{d}}_{i}}{{d}_{0}*{L}_{Target}})}^{2}}\right]$$

where:

- L_Target_ is the length of the chromosome.
- di is an instance of a distance value between two bins.
- L_ali_ Represents the count of all aligned residues.
- d_0_ is a normalizing parameter.

The TM-score is a metric to measure the structural similarity of two proteins or models [52, 53]. A TM-score value can be between (0,1] were 1 indicates two identical structures [52]. A score of 0.17 indicates pure randomness, and a score above 0.5 indicates the two structures have mostly the same folds [53]. Hence the higher, the better.

### Mean Squared Error (MSE)

MSE is defined below [54]:

$$dMSE= \frac{1}{n}*\sum {({d}_{i}-{D}_{i})}^{2}$$

where:

- ${D}_{i}$ and ${d}_{i}$ are instances of distance values from the data and another data source.
- The value n is the size of the data set.

### Huber loss

Huber loss is defined below [55]:

$$Huber Loss=\{ \begin{array}{cc}\frac{1}{2}*{d}^{2},& \left|d\right|\le \alpha \\ \alpha *\left(\left|d\right|-\frac{1}{2}*\alpha \right),& \left|d\right|> \alpha \end{array}$$

where:

- $d$ is an instance of distance values from the data and another data source
- $\alpha$ is a positive real number used to decide the transition point between the top and bottom loss functions. We varied $\alpha$ with values of 0.1, 0.5, and 0.9.

### Data

Our study used the yeast synthetic or simulated dataset from Adhikari et al., 2016 [27] to perform parameter tuning and validation. The simulated dataset was created from a yeast structure for chromosome 4 at 50 kb resolution [56]. The number of genome loci in the synthetic dataset is 610. We used the GM12878 cell Hi-C dataset to analyze a real dataset, GEO Accession number GSE63525 [57]. The normalized contact matrix was downloaded from the GSDB database with GSDB ID: OO7429SF [58].

### Parameters estimation

We used the yeast synthetic dataset to decide on ParticleChromo3D's best parameters. We used this data set to investigate the mechanism for choosing the best alpha conversion factor for input Hi-C data. Also, determine the optimal swarm size; determine the best threshold value for the algorithm, inertia value(w), and the best coefficients for our PSO velocity (${c}_{1}$ and ${c}_{2}$). We evaluated our reconstructed structures by comparing them with the synthetic dataset's true distance structure provided by Adhikari et al., 2016 [27]. We evaluated our algorithms with the DPCC, DSCC, DRMSE, and TM-score metrics. Based on the results from the evaluation, the default value for the ParticleChromo3D parameters are set as presented below:

### Conversion factor test ($\mathrm{\alpha }$)

The synthetic interaction frequency data set was generated from a yeast structure for chromosome 4 at 50 kb [38] with an $\alpha$ value of 1 using the formula: $IF=1/{D}^{\alpha }$. Hence, the relevance of using this test data is to test if our algorithm can predict the alpha value used to produce the synthetic dataset. For both DPCC and DSCC, our algorithm performed best at a conversion factor (alpha) of 1.0 (Additional file 1: Figure S1). Our algorithm's default parameter setting is that it searches for the best alpha value in the range [0.1, 1.5]. Side by side comparison of the true simulated data (yeast) structure and the reconstructed structure by ParticleChromo3D shows that they are highly similar (Additional file 1: Figure S2).

### Swarm size

The swarm size defines the number of particles in the PSO algorithm. We evaluated the performance of the ParticleChromo3D with changes in swarm size (Additional file 1: Figure S3A, S3B, S3C). Also, we evaluated the effect of an increase in swarm size against computational time (Additional file 1: Figure S3D). Our result shows that computational time increases with increased swarm size. Given the computational implication and the algorithm’s performance at various swarm size, we defined a swarm size of 15 as our default value for this parameter. According to our experiments, the Swarm size 10 is most suitable if the user’s priority is saving computational time, and swarm size 20 is suitable when the user's preference is algorithm performance over time. Hence, setting the default swarm size 15 gives us the best of both worlds. The structures generated by ParticleChromo3D also shows that the result at swarm size 15 (Additional file 1: Figure S4C) and 20 (Additional file 1: Figure S4D) are most similar to the simulated data true structure represented in Additional file 1: Figure S2A.

### Threshold: optimal parameter to determine structure stability

The threshold parameter is designed to serve as an early stopping criterion if the algorithm converges before the maximum number of iterations is reached. Hence, we evaluated the effect of varying threshold levels using the evaluation metrics (Additional file 1: Figure S5). The output structures generated by each threshold also allow a visual examination of a threshold value (Additional file 1: Figure S6). We observed that the lower the threshold, the more accurate (Additional file 1: Figure S5) and similar the structure is to the generated true simulated data structure in Additional file 1: Figure S2A (Additional file 1: Figure S6F). It worth noting that this does have a running time implication. Reducing the threshold led to a longer running time. However, since this was a trade-off between a superior result and longer running time or a reasonably good result and short running time, we chose the former for ParticleChromo3D. The default threshold for our algorithm is 0.000001. We used this control parameter.

### Confidence coefficient (${c}_{1}$ and ${c}_{2}$)

The $${c}_{1}$$ and $${c}_{2}$$ parameters represent the local-confidence and local and global swarm confidence level coefficient. Kennedy and Eberhart, 1995 [41] proposed that $${c}_{1}$$= $${c}_{2}=2$$. We experimented with testing how this value's changes affected our algorithm's accuracy for local confidence coefficient ($${c}_{1})$$ 0.3 to 0.9 and global confidence values 0.1 to 2.8 (Additional file 1: Figure S9 and S10). From our results, we found that a local confidence coefficient ($${c}_{1})$$ of 0.3 with a global confidence coefficient ($${c}_{2}$$) of 2.5 performed best (Additional file 1: Figure S7). Hence, these values were set as ParticleChromo3D's confidence coefficient values. The accuracy results generated for all the local confidence coefficient (c1) at varying global confidence values is compiled in Additional file 1: Figure S8.

### Random numbers (${R}_{1}$ and ${R}_{2}$)

${R}_{1}$ and ${R}_{2}$ are uniform random numbers between 0 and 1 [59].

## Results

### Assessment on simulated data

**Fig. 4:**
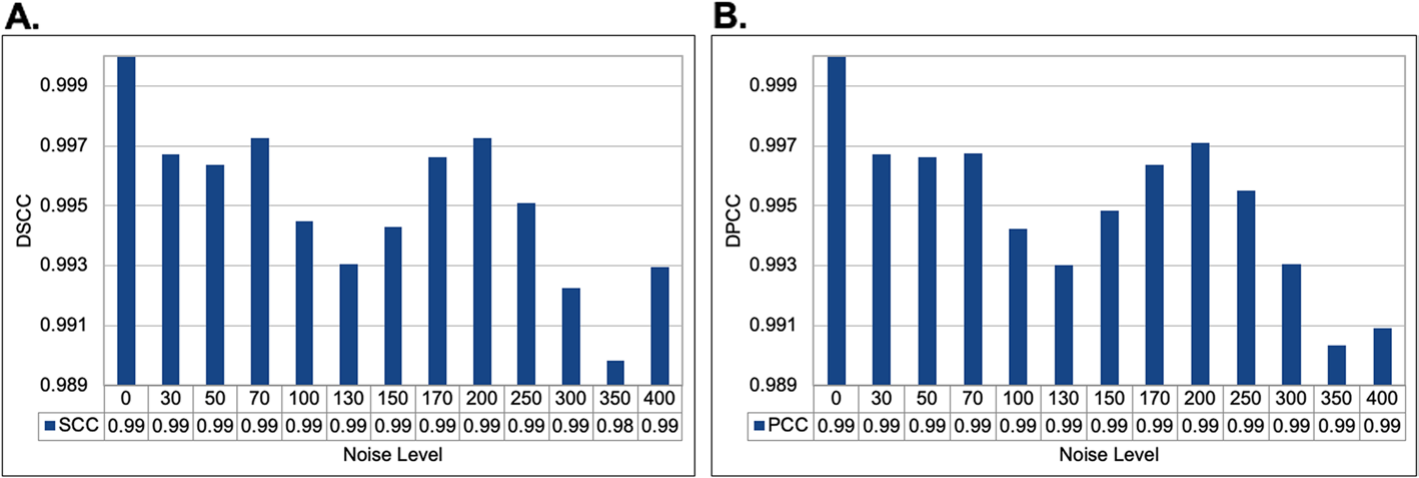
Assessment of the structures generated by ParticleChromo3D for the simulated dataset on varying noise levels. **A** A plot of the DSCC versus Noise level. **B** A plot of DPCC versus the Noise level. This plot shows the DSCC and DPCC accuracy of the structures generated by ParticleChromo3D at different noise levels introduced. In Fig. 4A and Fig. 4B, the Y-axis denotes the metric score in the range [-1,1]. The X-axis denotes the Noise level. A higher DSCC and DPCC value is better

We evaluated how noise levels affect ParticleChromo3D's ability to predict chromosome 3D structures in the presence of noise. Using the yeast synthetic dataset from Adhikari et al., 2016 [27]. The data were simulated with a varying noise level. Adhikari, et al. introduced noise into the yeast IF matrix to make 12 additional datasets with different levels of noise at 3%, 5%, 7%, 10%, 13%, 15%, 17%, 20%, 25%, 30%, 35%, and 40%. As reported by the authors, converting this IF to their distance equivalent produced distorted distances that didn’t match the true distances. They were thereby simulating the inconsistent constraints that can sometimes be observed in un-normalized Hi-C data. As shown, our algorithm performed the best with no noise in the data at 0 (Fig. 4).

**Fig. 5:**
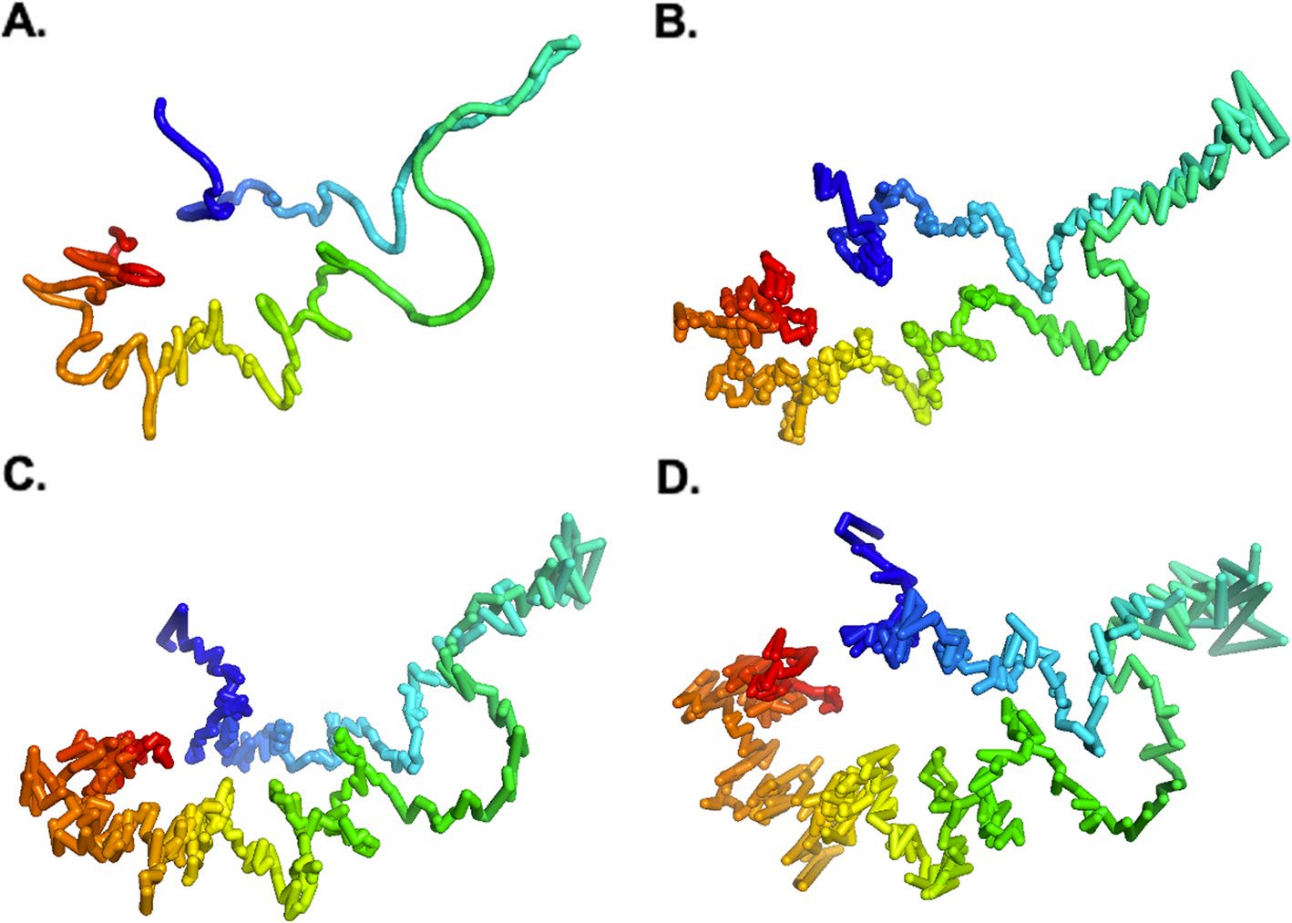
Structures generated by ParticleChromo3D at different Noise levels. Here, we show the structure generated by ParticleChromo3D at Noise level = **A** 0, that is no Noise, **B** 7% (70), **C** 20% (200), and **D** 40% (400)

Furthermore, the other result obtained by comparing the ParticleChromo3D algorithm’s output structure from the noisy input datasets with the simulated dataset’s true structure shows that it can achieve a competitive result when dealing with un-normalized or noisy Hi-C datasets (Fig. 5). The result shows that our algorithm can achieve the results obtainable at reduced noise level even at increased noise as indicated by Noise 7% (Fig. 5B) and 20% (Fig. 5C), respectively (Fig. 4). Also, the difference in performance between the best structure and the worst structure is ~ 0.01. Hence, our algorithm cannot potentially be affected by the presence of noise in the input Hi-C data.

### Assessment on real Hi-C data

**Fig. 6:**
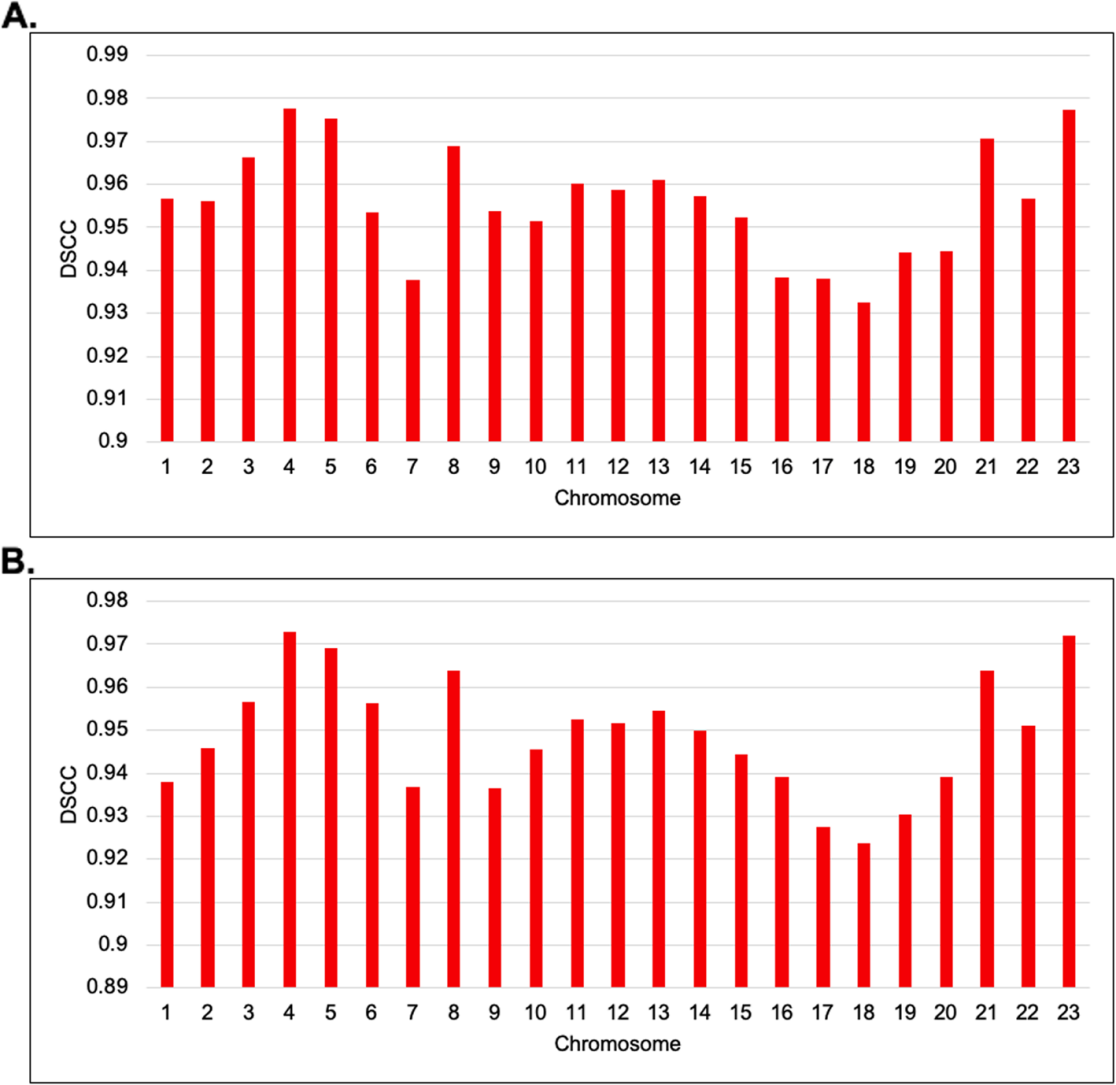
Performance evaluation of ParticleChromo3D using DSCC values for 1 MB and 500 KB resolution GM12878 cell Hi-C data. **A** A plot of ParticleChromo3D DSCC performance on 1 MB GM12878 cell Hi-C data chromosome 1 to 23 **B** A plot of ParticleChromo3D DSCC performance on 500 KB GM12878 cell Hi-C data for chromosome 1 to 23

For evaluation on the real Hi-C data, we used the GM12878 B-lymphoblastoid cells line by Rao et al., 2014 [57]. The normalized 1 MB and 500 KB resolution interaction frequency matrices GM12878 cell line datasets were downloaded from the GSDB repository under the GSDB ID OO7429SF [58]. The datasets were normalized using the Knight-Ruiz normalization technique [16]. The performance of ParticleChromo3D was determined by computing the DSCC value between the distance matrix of the normalized frequency input matrix and the Euclidean distance calculated from the predicted 3D structures. Figure 6 shows the assessment of ParticleChromo3D on the GM12878 cell line dataset. The reconstructed structure by ParticleChromo3D is compared against the input IF expected distance using the DPCC, DSCC, and RMSD metrics for the 1 MB and 500 KB resolution Hi-C data. When ParticleChromo3D performance is evaluated using both 1 MB and 500 KB resolution HiC data of the GM12878 cell, we observed some consistency in the algorithm’s performance for both datasets. Chromosome 18 had the lowest DSCC value of 0.932 and 0.916 at 1 MB and 500 KB resolutions, respectively, while chromosome 5 had the highest DSCC value of 0.975 and 0.966 at 1 MB and 500 KB resolutions, respectively.

### Model consistency: robustness test over number of independent runs

**Fig. 7:**
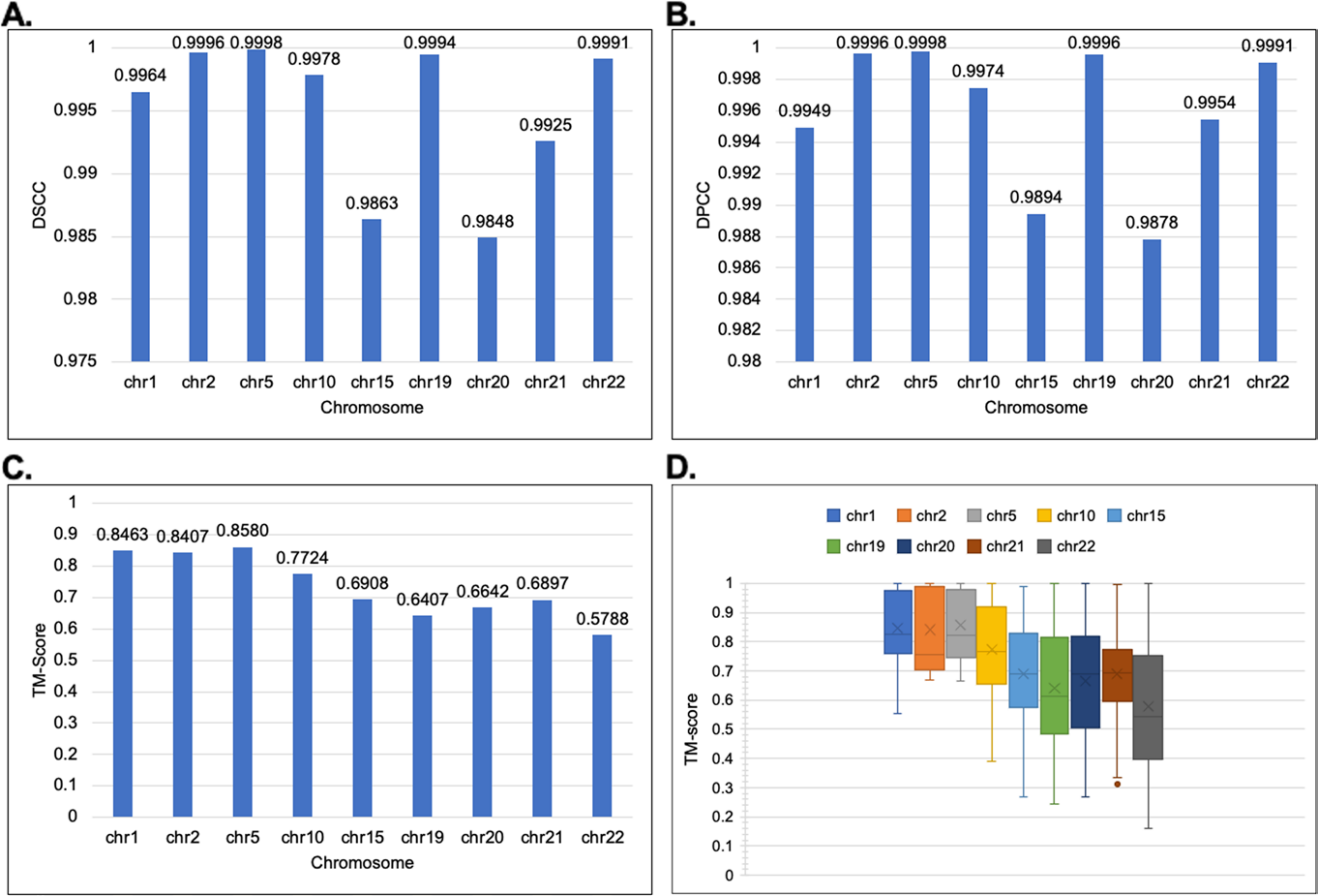
The model consistency check for 1 MB resolution structures generated by ParticleChromo3D using different evaluation metrics. **A** The average DSCC Between 30 Structures per chromosome at 1 MB Resolution for the GM12878 datasets. **B** The average DPCC Between 30 Structures per chromosome at 1 MB Resolution for the GM12878 datasets. **C** The average TM-Score Between 30 Structure per chromosome at 1 MB Resolution for the GM12878 datasets. **D** The boxplot shows the distribution of the 30 structure's TM-score by chromosome for the GM12878 datasets. The Y-axis denotes the DSCC and DPCC metric score in the range [-1,1], and TM-Score in the range [-0,1]. The X-axis denotes the chromosome. A higher DSCC, DPCC, and TM-Score value is better

**Fig. 8:**
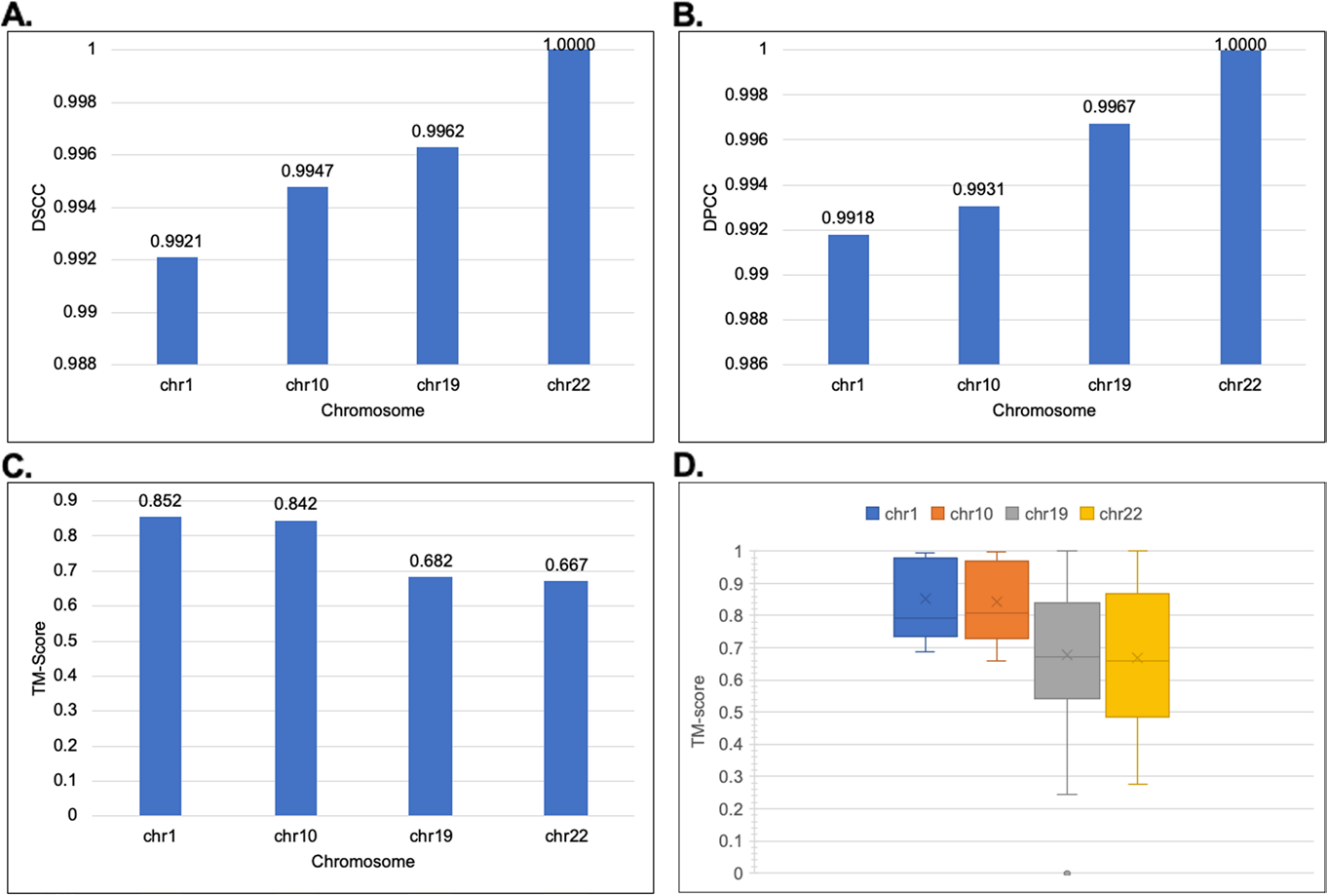
The model consistency check for 500 KB resolution structures generated by ParticleChromo3D using different evaluation metrics. **A** The average DSCC Between 30 Structures per chromosome at 500 KB Resolution for the GM12878 datasets. **B** The average DPCC Between 30 Structures per chromosome at 500 KB Resolution for the GM12878 datasets. **C** The average TM-Score Between 30 Structure per chromosome at 500 KB Resolution for the GM12878 datasets. **D** The boxplot shows the distribution of the 30 structure's TM-score by chromosome for the GM12878 datasets. The Y-axis denotes the DSCC and DPCC metric score in the range [-1,1], and TM-Score in the range [-0,1]. The X-axis denotes the chromosome. A higher DSCC, DPCC, and TM-Score value is better

Next, we assessed the consistency of our generated structures. We created 30 structures for the chromosomes and then evaluated the structure’s similarity using the DSCC, DPCC, DRMSE, and TM-Score (Fig. 7). We assessed the consistency for both the 1 MB and 500 KB resolution Hi-C data of the GM12878 cell. As illustrated for the TM-score, a score of 0.17 indicates pure randomness, and a score above 0.5 indicates the two structures have mostly the same folds. Hence the higher, the better. Our results show from the selected chromosomes that the structures generated by ParticleChromo3D are highly consistent for both the 1 MB (Fig. 7) and 500 KB (Fig. 8) datasets. As shown in Fig. 7 for the 1 MB Hi-C datasets, the average DSCC and DPCC values recorded between the models for the selected chromosomes is >  = 0.985 and >  = 0.988, respectively, indicating that chromosomal models generated by ParticleChromo3D are highly similar. It also indicates that it finds an absolute 3D model solution on each run of the algorithm (Fig. 7C and Fig. 7D). Similarly, as shown in Fig. 8, for the 500 KB Hi-C datasets, the average DSCC and DPCC values recorded between the models for the selected chromosomes is >  = 0.992.

### Comparison with existing chromosome 3D structure reconstruction methods

**Fig. 9:**
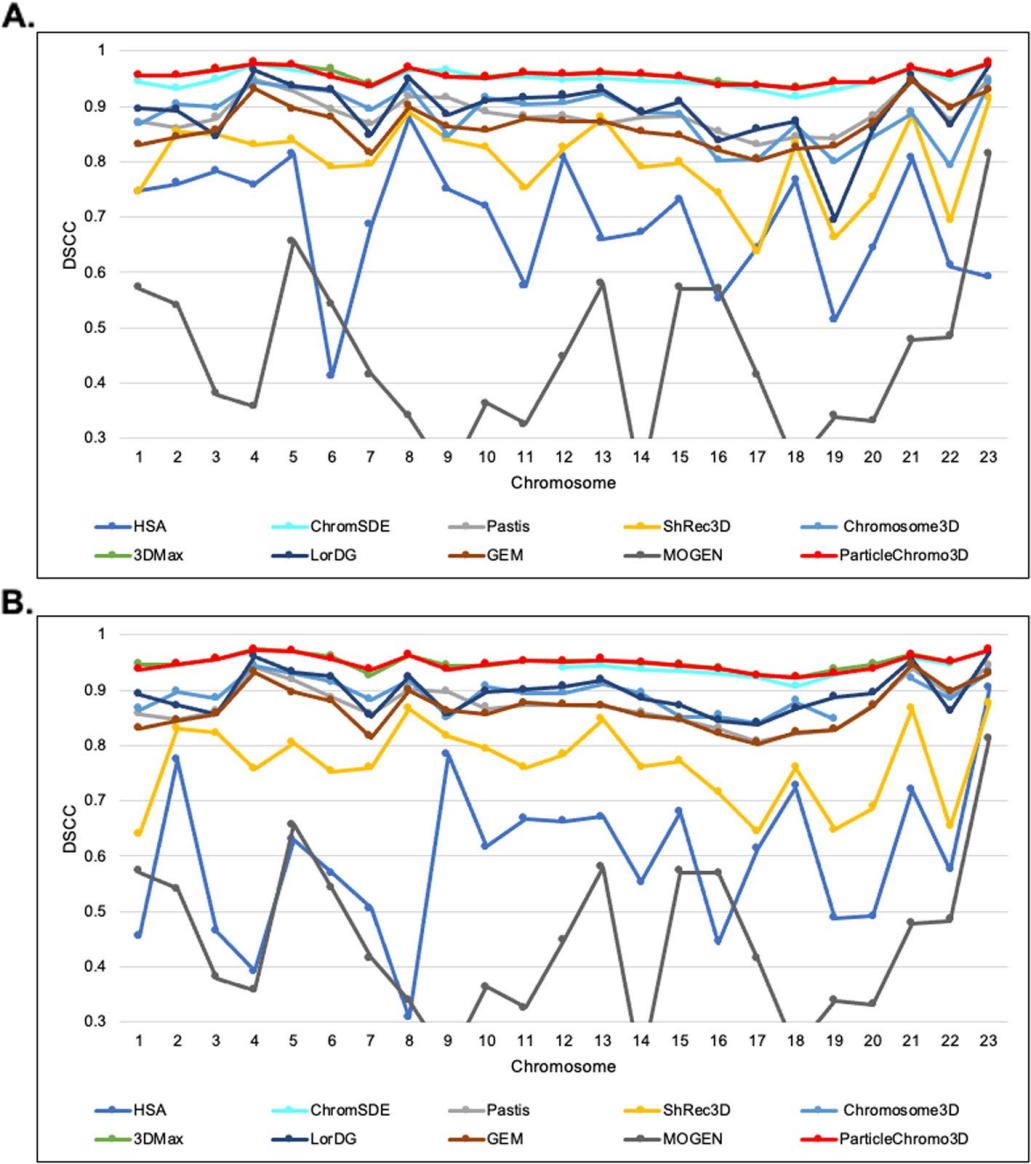
A comparison of the accuracy of nine existing methods and ParticleChromo3D for 3D structure reconstruction on the 1 MB and 500 KB real Hi-C dataset. **A** An DSCC Comparison of 3D structure reconstruction methods on the GM12878 Hi-C dataset at 1 Mb resolution for chromosomes 1 to 23. **B** A DSCC Comparison of 3D structure reconstruction methods on the GM12878 Hi-C dataset at 500 KB resolution for chromosomes 1 to 23. The Y-axis denotes the DSCC metric score in the range [-1,1], and X-axis denotes the chromosome. A higher DSCC value is better

Here, we compared the performance of ParticleChromo3D side by side with nine existing high-performing chromosome 3D structure reconstruction algorithms on the GM12878 data set at both the 1 MB and 500 KB resolutions. The reconstruction algorithms are ChromSDE [25], Chromosome3D [27], 3DMax [28], ShRec3D [29], LorDG [30], HSA [32], MOGEN [34], GEM [38] and PASTIS [40] (Fig. 9). According to the DSCC value reported, we observed that ParticleChromo3D outperformed most of the existing methods in many chromosomes evaluated at 1 MB and 500 KB resolution. At a minimum, ParticleChromo3D secured the top-two best overall performance position among the ten algorithms compared. ParticleChromo3D achieving these results against these methods and algorithms shows the robustness and suitability of the PSO algorithm to be used to solve the 3D chromosome and genome structure reconstruction problem.

## Discussion

### Parameter optimization: swarm size versus time

We discussed the Swarm Size value's relevance in the *Parameters Estimation* section. We showed on the synthetic dataset that a Swarm Size value of 5 did not produce satisfactory performance. However, it was the fastest considering the other swarm sizes. At SS = 10, the performance was significantly improved than at SS = 5, but with an increase in computation time as a consequence. SS values 15 and 20 similarly achieved better performance, but the cost of this performance improvement similarly is an increase in the program running time. However, we settled for a SS = 15 because it achieved one of the best performances, and the computational cost can be considered manageable. To investigate the implication of our choice, we carried out two tests discussed below:

### ParticleChromo3D performance on different swarm size values

**Fig. 10:**
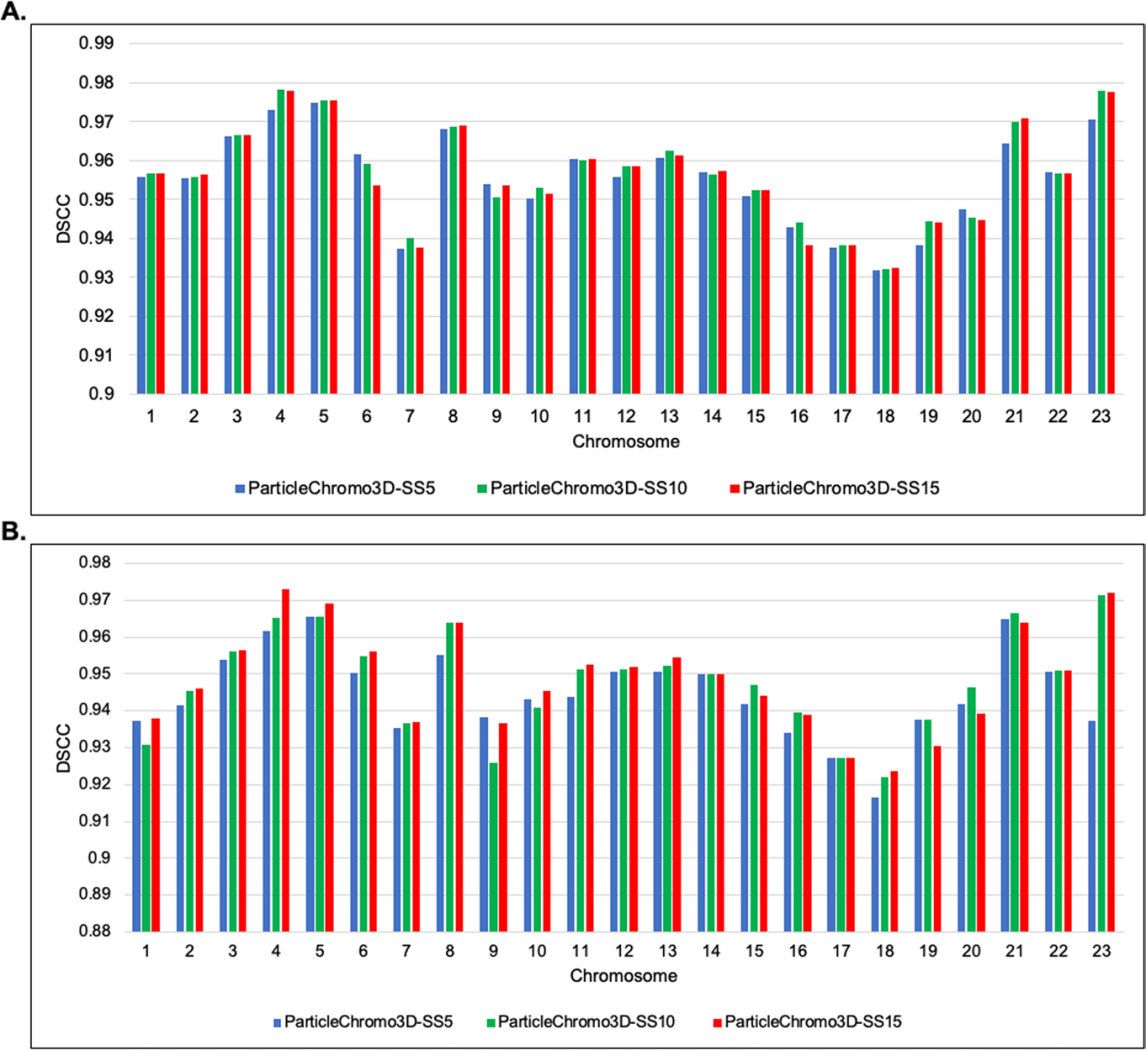
ParticleChromo3D DSCC performance on Swarm Size values 5,10 and 15 for 1 MB and 500 KB GM12878 cell Hi-C data. **A** Comparing the performance by ParticleChromo3D on the 1 MB GM12878 cell Hi-C data at Swarm Size values 5, 10, and 15. **B** Comparing the performance by ParticleChromo3D on the 500 KB GM12878 cell Hi-C data at Swarm Size values 5, 10, and 15. The Y-axis denotes the DSCC metric score in the range [-1,1], and X-axis denotes the chromosome. A higher DSCC value is better

First, we evaluated the performance of the ParticleChromo3D algorithm on the GM12878 data set on both the 1 MB and 500 KB resolutions at Swarm Sizes 5, 10, and 15 to ensure that the performance at SS = 15 that we observed on the synthetic dataset is carried over to the real dataset (Fig. 10). The 1 MB and 500 KB dataset result shows that SS = 15 achieved the best DSCC value mostly across the chromosomes (Fig. 10). However, we observed that the result generated at SS = 10 were also competitive and achieved an equal performance a few times with SS = 15. This shows us that choosing the SS = 10 does not necessarily reduce the performance of our ParticleChromo3D. There is an additional gain of saving on computational time if this value is used.

### Computational time

Second, we evaluated the time it took our algorithm to perform the 3D reconstruction for select chromosomes of the 1 MB and 500 KB GM12878 cell Hi-C data set. The modeling of the structures generated by ParticleChromo3D for the synthetic and real dataset was done on an AMD Ryzen 7 3800 × 8-Core Processor, 3.89GHZ with installed RAM 31.9 GB.

**Fig. 11:**
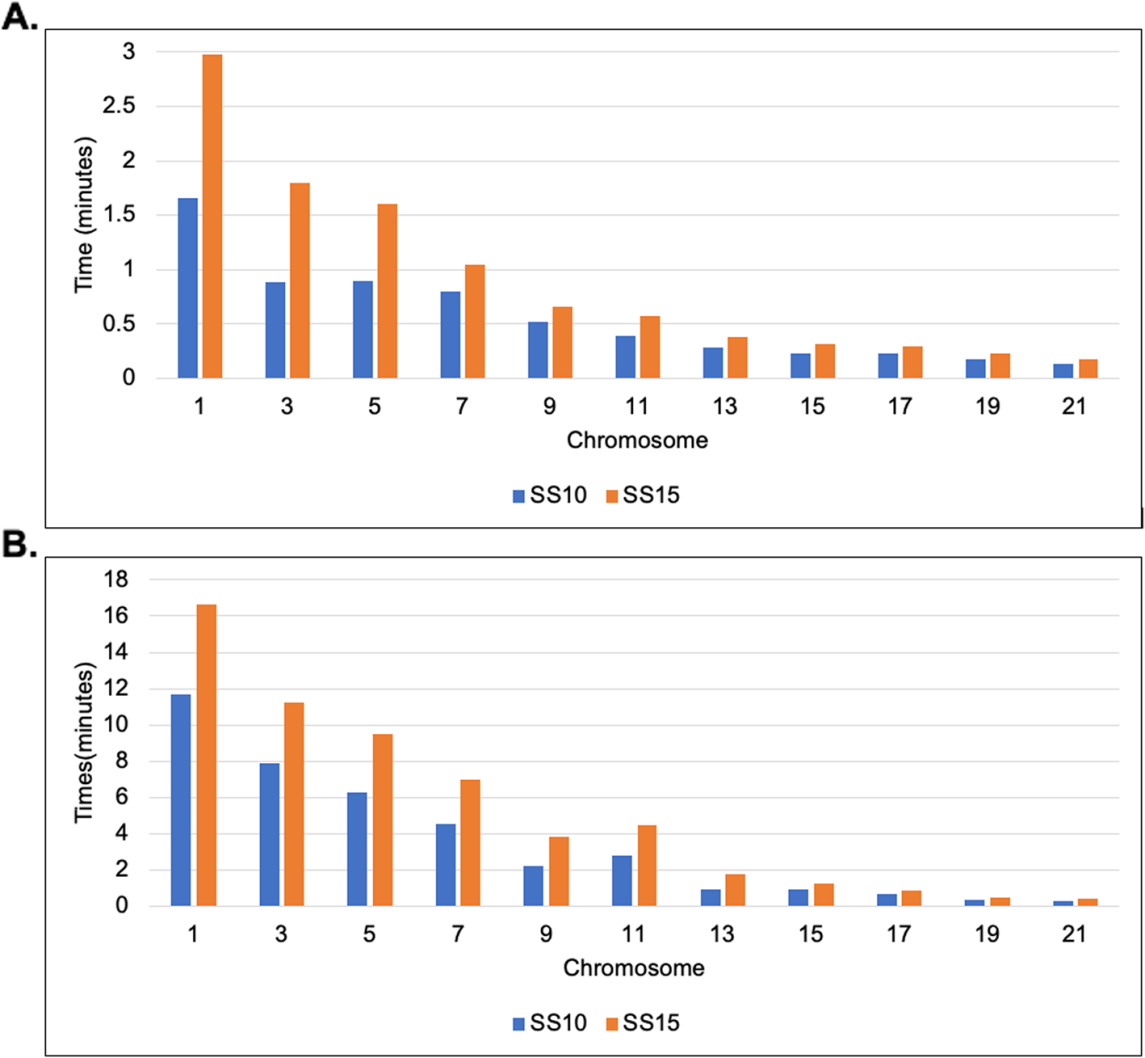
ParticleChromo3D Computational Time at Swarm Size (SS) 10 and 15 for 1 MB and 500 KB GM12878 cell Hi-C data. **A** Comparing the running time for ParticleChromo3D for select chromosomes for 1 MB GM12878 cell Hi-C data (**B**) A comparison of the running time for ParticleChromo3D for select chromosomes for 500 KB GM12878 cell Hi-C data. The Y-axis denotes the running time for ParticleChromo3D in minutes, and X-axis denotes the chromosome

ParticleChromo3D is programmed to multithread. It utilizes each core present on the user's computer to run a specific task, speeding up the modeling process and significantly reducing computational time. Accordingly, the more the number of processors a user has, the faster ParticleChromo3D will generate an output 3D structures. As mentioned earlier in the Parameter Estimation section, one of the default settings for ParticleChromo3D is to automatically determine the best conversion factor that fits the data in the range [0.1, 1.5]. Even though this is one of our ParticleChromo3D's strengths, this process has the consequence of increasing the algorithm's computational time. Based on the real Hi-C dataset analysis, our result shows that the Swarm Size 10 consistently has a lower computational time than the SS = 15 as speculated for the 500 KB and 1 MB Hi-C datasets (Fig. 11). These results highlight an additional strength of ParticleChromo3D that it can achieve a competitive result in a lower time (Fig. 11) without trading it off with performance (Fig. 10). It is worth noting that we recommend that users can set the Swarm Size to the preferred value depending on the objective. In this manuscript, we favored the algorithm achieving a high accuracy over speed. We made up for this by making our algorithm multi-threaded, reducing the running time significantly.

### Loss functions

A recurring question that occurred in this work is the impact of the loss function choice on the performance of the algorithm. To address this, we performed experiments on the simulated datasets and the real Hi-C datasets using the following loss functions: Sum of Squared Error (SSE), Mean Squared Error (MSE) [54], Root Mean Squared Error, and the Huber loss [55].

**Fig. 12:**
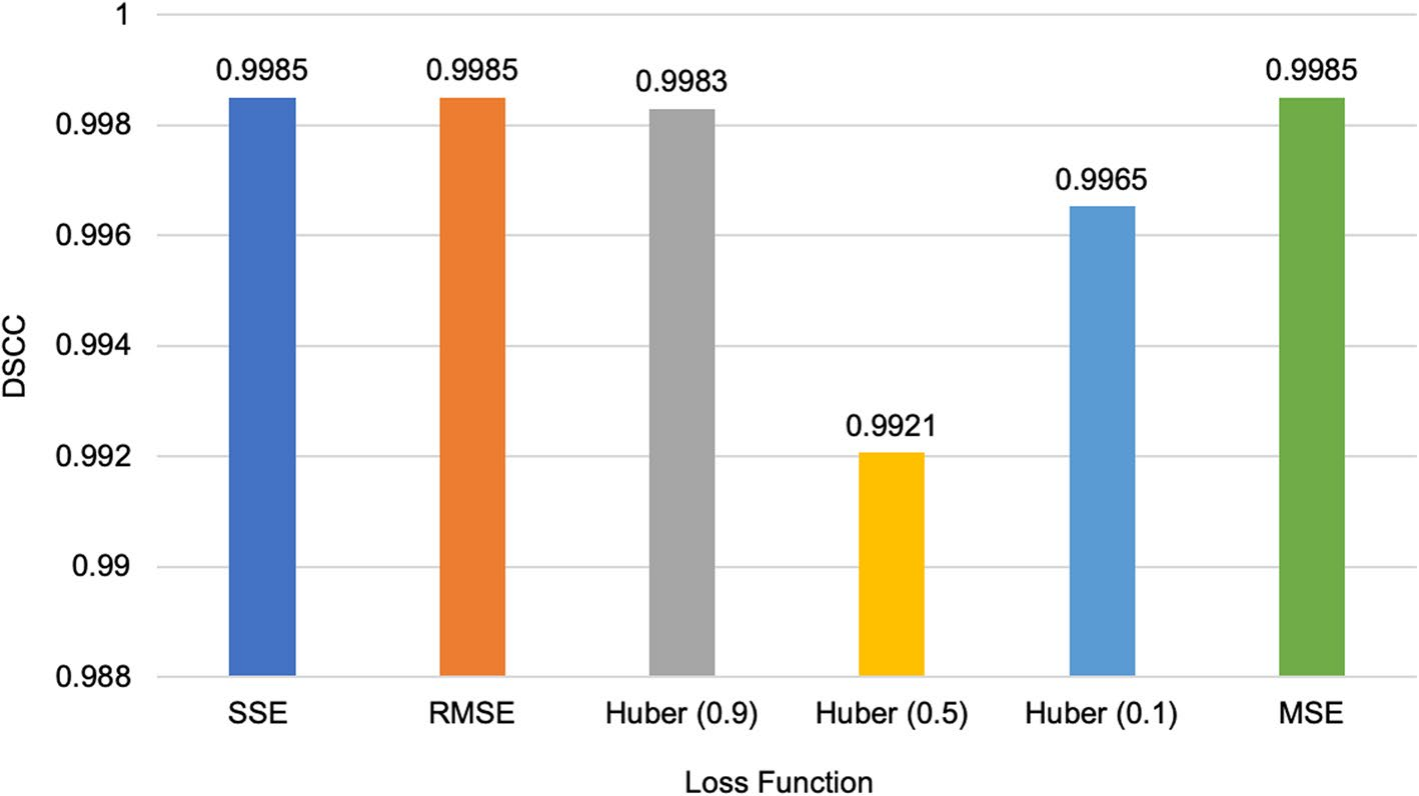
Loss function versus DSCC on a simulated dataset. A side-by-side comparison of DSCC performance at loss functions Sum of Squared Error (SSE), Mean Squared Error (MSE), Root Mean Squared Error (RMSE), and Huber loss on the simulated dataset. The Y-axis denotes the DSCC metric score in the range [-1,1], and X-axis denotes the loss function. A higher DSCC value is better

**Fig. 13:**
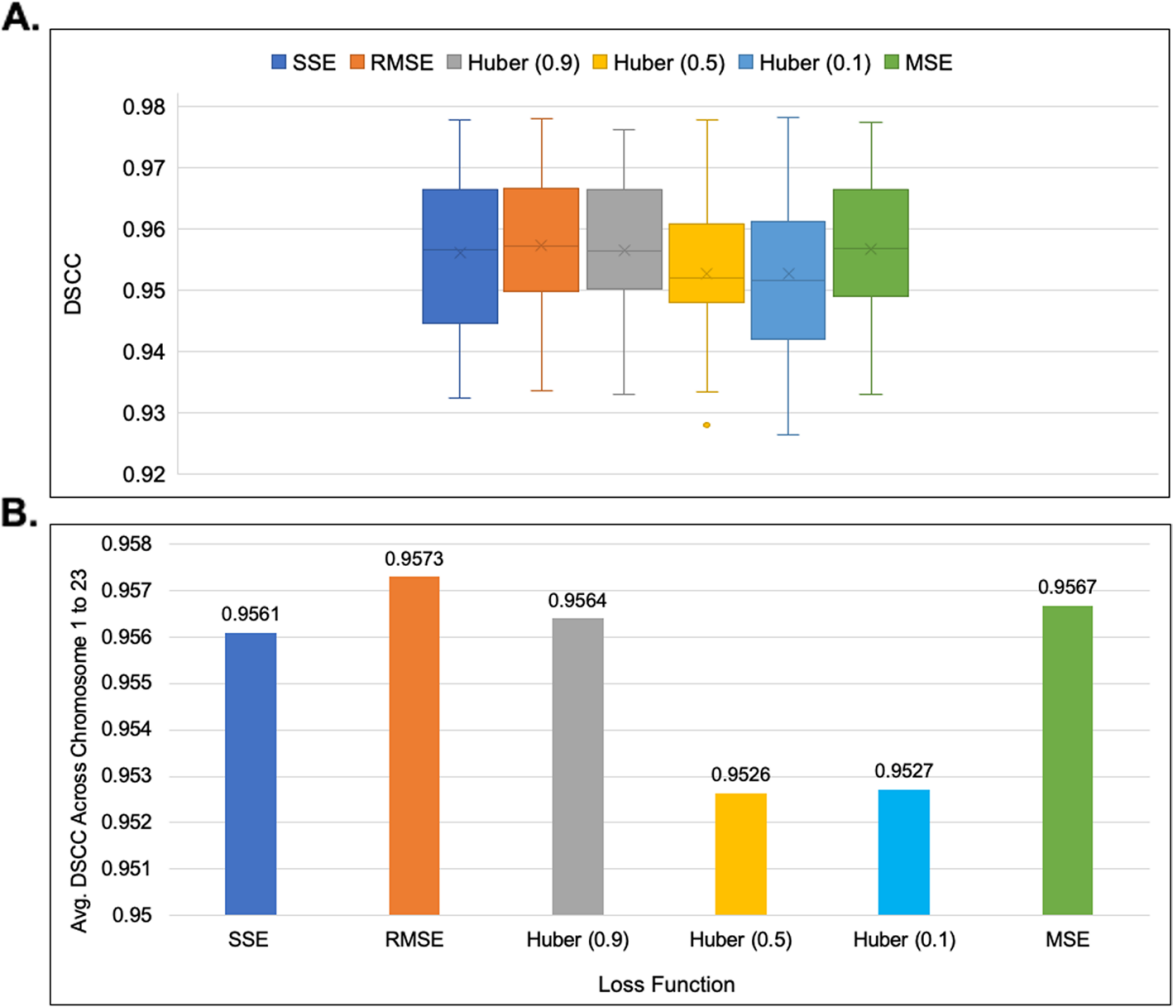
Comparison of Loss Function distribution for 1 MB GM12878 cell Hi-C data. **A** A boxplot showing the DSCC value distribution of `3D output structure of 1 MB GM12878 cell Hi-C data chromosome 1 to 23 obtained by using loss functions SSE, RMSE, Huber-0.9, Huber -0.5, Huber-0.1, and MSE **B** A Comparison of the average DSCC value of 1 MB GM12878 cell Hi-C data chromosome 1 to 23 obtained by using loss functions SSE, RMSE, Huber-0.9, Huber -0.5, Huber-0.1, and MSE. The Y-axis denotes the DSCC metric score in the range [-1,1], and X-axis denotes the loss function. A higher DSCC value is better

**Fig. 14:**
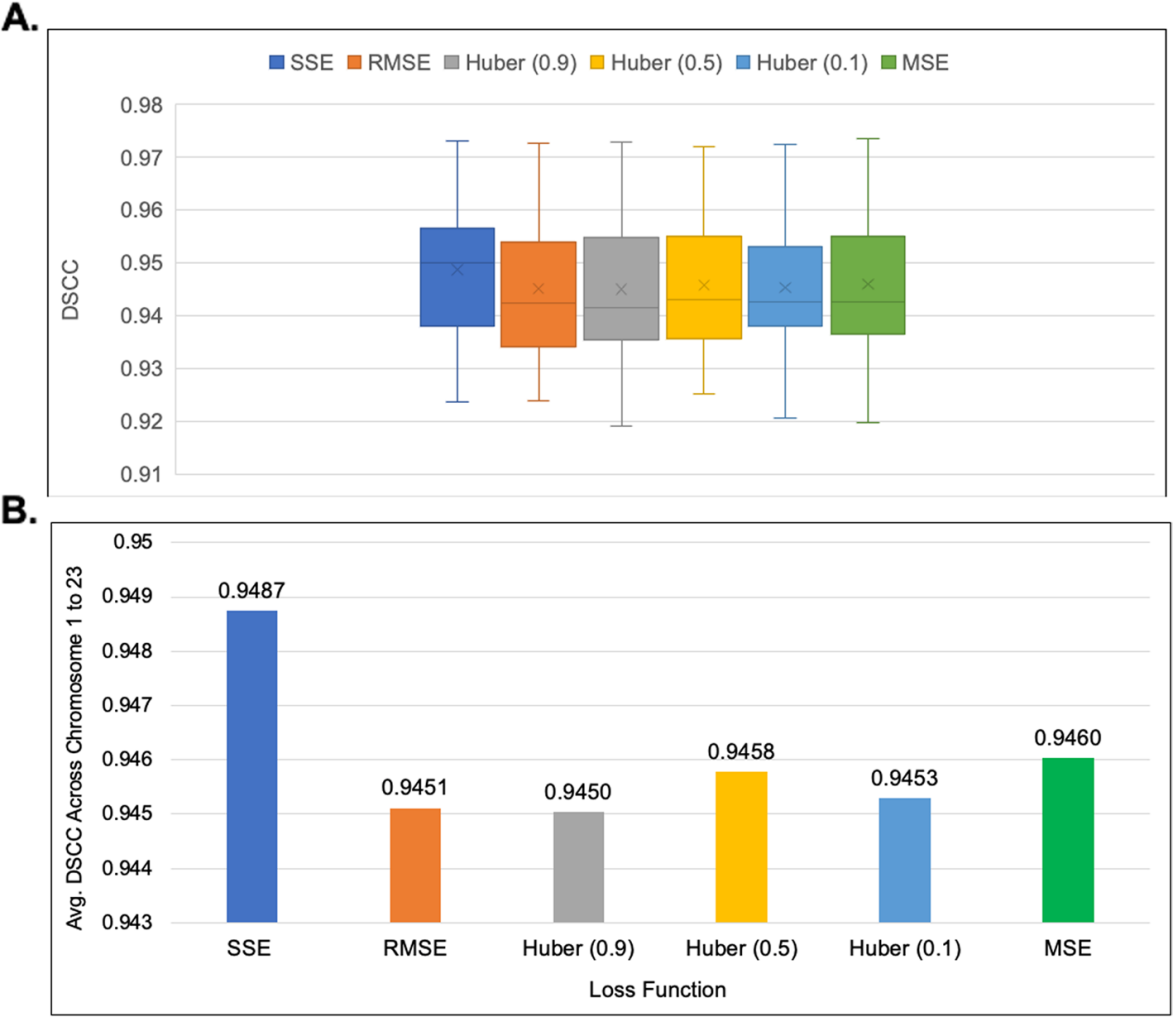
Comparison of Loss Function distribution for 500 KB GM12878 cell Hi-C data. **A** A boxplot showing the DSCC value distribution of `3D output structure of 500 KB GM12878 cell Hi-C data chromosome 1 to 23 obtained by using loss functions SSE, RMSE, Huber-0.9, Huber -0.5, Huber-0.1, and MSE (**B**) A Comparison of the average DSCC value of 500 KB GM12878 cell Hi-C data chromosome 1 to 23 obtained by using loss functions SSE, RMSE, Huber-0.9, Huber -0.5, Huber-0.1, and MSE. The Y-axis denotes the DSCC metric score in the range [-1,1], and X-axis denotes the loss function. A higher DSCC value is better XYZ coordinates

ParticleChromo3D had the best performance on the simulated dataset when the SSE, RMSE, and MSE loss functions were used (Fig. 12). These three loss functions also reported the same performance. As the representative loss function to use in our experiments, we chose the SSE loss function for all the results reported. However, we had the underlying question if this performance can be replicated on the real Hi-C dataset. Hence, we performed the loss function test on the real Hi-C dataset to check the impact of the loss function on the performance of our algorithm. On the 1 MB dataset, the RMSE loss function reported the best performance; it had a comparatively short box plot with a high median, suggesting that the DSCC values across the chromosomes are closer to each other (Fig. 13). On the 500 KB Hi-C dataset, the SSE loss function showed a better performance than the other loss functions (Fig. 14). From this test, we observed that, though minimal, the choice of the loss function had some impact on the algorithm’s result. Hence, we included an option in the ParticleChromo3D tool for users to select the loss function to use for their 3D reconstruction problem from the options above.

**Fig. 15:**
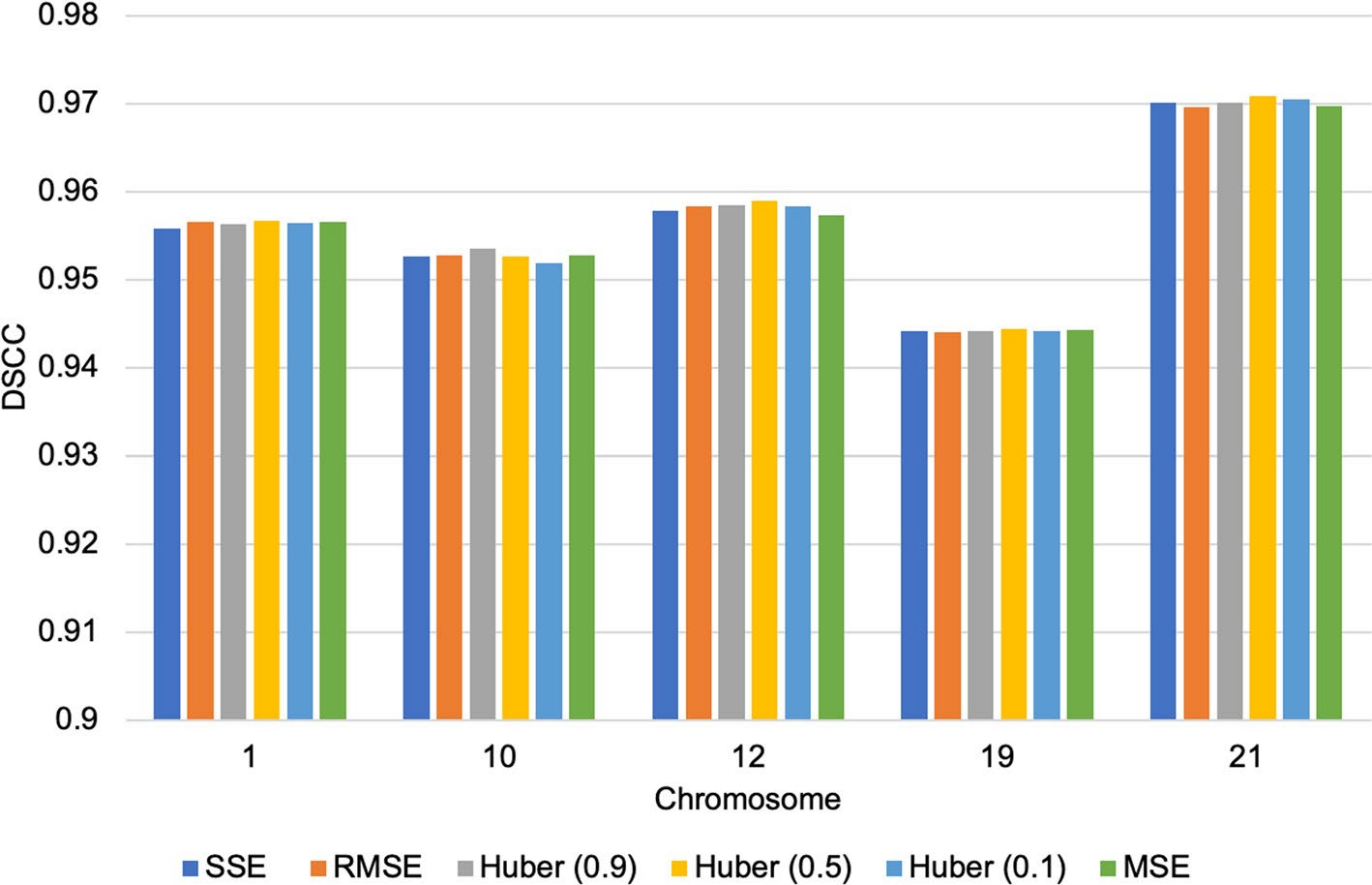
Loss Function comparison without random XYZ coordinate initialization. We used the same XYZ coordinates for the initialization across the loss function run execution for each of the chromosomes 1,10,12,19, and 21 of 1 MB GM12878 cell Hi-C data. With this configuration, we had the same initialization for the algorithm to use for the 3D structure reconstruction for the loss functions SSE, RMSE, Huber-0.9, Huber -0.5, Huber-0.1, and MSE. The Y-axis denotes the DSCC metric score in the range [-1,1], and X-axis denotes the chromosomes. A higher DSCC value is better

Finally, we had a conjecture that a contributing factor to loss function difference in performance was from the impact of the randomized initial XYZ coordinate assigned to each loss function job execution. We mean that because each loss function on different runs gets a different randomized XYZ coordinate assignment, it affects the algorithm’s convergence and performance. To test this assumption, instead of using a randomized XYZ coordinate for the loss function test, we used the same XYZ coordinate for the initialization across the loss functions test for 1 MB GM12878 cell Hi-C data chromosomes 1,10,12,19, and 21. We observed that with this configuration, ParticleChromo3D’s performance across the loss functions was more stable and consistent (Fig. 15). Hence, confirming the conjecture that each of the loss functions by themselves have little impact on performance, but an algorithm’s performance could be influenced, though minimally (Fig. 13 and Fig. 14), by the randomized initial XYZ coordinate assignment done at the initialization stage (Fig. 15).

### Topologies

So far, we have described PSO through the lens of a global best optimizer with a randomly initiated topology. While this implementation was the focus of this paper, we also tested if we could use our optimized hyperparameters on other topologies and see even better results. The open-source library we used was PySwarms–an extensible research toolkit for PSO in Python [60] and we implemented its topologies local best optimization with a ring topology, pyramid, star, and random. We wrote an adapter that converted the frequency data into a format that PySwarms could ingest and optimize. We examined these topologies using a swarm size of 15 as justified by our validations in the *Parameter Estimation* section above. Each of these PSO algorithm topologies are described and compared with our results below:

### Local best optimization with ring topology

Local best is like global best in that it finds a set of potential solutions and then update position and velocity [61]. The key difference is that it uses a ring topology (see Additional file 1: Figure S11) on initialization that causes particle to be attracted to their neighbors. PySwarms uses a k-D tree to select its neighbors. The velocity equation is [41, 61] shown as in Eqs. (3) and (4):

3 $${V}_{i,n+1}=w*{V}_{ij,n}+ {c}_{1}* {R}_{j,1}*\left( {P}_{ij,n}- {X}_{ij,n}\right)+ {c}_{2}* {R}_{j,2}*\left( {G}_{j,n}- {X}_{ij,n}\right)$$

Then position is updated as follow:

4 $${X}_{i,n+1}= {X}_{i,n}+ {V}_{i,n+1}$$

where:

- ${V}_{i,n}$ is the current velocity at iteration $n$ of particle i.
- ${c}_{1}$ and ${c}_{2}$ are two real numbers that stand for local and global weights and are the personal best of the specific particle and the global best vectors, respectively, at iteration $n$ [61].
- The ${R}_{j,1}$ and ${R}_{j,2}$ values are randomized values used to increase the explored terrain based off particle i’s neighbors [61].
- $w$ is the inertia weight parameter, and it determines the rate of contribution of a velocity [41].
- ${G}_{j,n}$ represents the best position of the particle’s neighbors at iteration $n$.
- ${P}_{ij,n}$ represents the best position of a particle and its neighbors.
- ${X}_{ij,n}$ is the best position of an individual particle at the iteration $n$.

Local Best has two additional hyperparameters that we will not be optimizing in the scope of this paper. These parameters are number of neighbors to consider and whether to measure distance using sum-of-absolute values or Euclidean distance. We chose to use Euclidean distance and three neighbors.

### Pyramid topology

The pyramid topology attempts to create spatially meaningful neighbors [62]. Pyramid does this by optimizing heuristics that implement natural neighbors using Delaunay Triangulation [62]. This algorithm works by first, randomly initializing the swarm [62]. Then it loops through the following steps for each particle until the end criterion is met [62]:

i. Compute the Delaunay.
ii. Seeing if the particles new position (derived from the Delaunay) is better than its old position.
iii. choosing its best neighbor
iv. Updating position and velocity with regards to its neighbors.

### Star topology

Star topologies are often used in conjunction with Global Best strategies with the main difference being that instead of a randomized or ring laydown on initialization they are generated to a star [63]. This looks like a ring with every ring connected to a particle in the middle which it considers its neighbor as shown in Additional file 1: Figure S12 [63]. It can use either the base global best velocity and position equations or it can use local best [63]. If it uses local best, it considers its neighbor the center point [63].

### Random topology

In a random topology the neighbors of a swarm are selected at random, and the starting positions can also be random [63]. The implementation we used uses the Dijkstra algorithm to find the distance of one particle to another and then if any unconnected particles exist add edges between them [63]. The Random Topology used the same additional hyper parameters as Local Best.

### Comparison of ParticleChromo3D’s algorithm topology with Other PSO Topologies

Here we compared ParticleChromo3D using the global best topology to local, pyramid, star, and random topologies described above. Due to computational time complexity, we experienced for these algorithms as the number of bins in the chromosomes increased, we only compared chromosomes 16–23 of the GM12878 data set. Overall, while the other topologies have their strengths, however, according to our results for this 3D structure prediction problem, the PSO implementation used in ParticleChromo3D algorithm–global best optimization with a random topology–outperformed the other algorithms. As shown in Additional file 1: Figure S13, ParticleChromo3D significantly outperformed the other topologies to the point that its worst run was better than any of the other topologies best runs.

## Conclusions

We developed a new algorithm for 3D genome reconstruction called ParticleChromo3D. ParticleChromo3D uses the Particle Swarm Optimization algorithm as the foundation of its solution approach for 3D chromosome reconstruction from Hi-C data. The results of ParticleChromo3D on simulated data show that with the best-fine-tuned parameters, it can achieve high accuracy in the presence of noise. The computational complexity of ParticleChromo3D may make it worthwhile to optimize this algorithm with GPU support or in a faster language than Python. We compared ParticleChromo3D accuracy with nine (9) existing high-performing methods or algorithms for chromosome 3D structure reconstruction on the real dataset. The results show that ParticleChromo3D is effective and a high performer by achieving more accurate results over the other methods in many chromosomes; and securing the top-two best overall position in our comparative analysis with different algorithms. Our experiments also show that ParticleChromo3D can also achieve a faster computational run time without losing accuracy significantly. ParticleChromo3D’s parameters have been optimized to achieve the best result for any input Hi-C by searching for the best conversion factor ($\alpha$) and using the optimal PSO hyperparameters for any given input automatically. This algorithm was implemented in python and can be run as an executable or as a Jupyter Notebook found at https://github.com/OluwadareLab/ParticleChromo3D

## Supplementary Information

**Additional file 1.**

## Data Availability

All real Hi-C data files are available from the GSDB database (accession number(s) OO7429SF. The models generated, all the datasets used for all analysis performed, and the source code for ParticleChromo3D are available at https://github.com/OluwadareLab/ParticleChromo3D

## Abbreviations

3C: Chromosome Conformation Capture
IF: Interaction Frequency
KR: Knight-Ruiz
PSO: Particle Swarm Optimization
SS: Swarm Size
DSCC: Distance Spearman Correlation Coefficient
DPCC: Distance Pearson Correlation Coefficient
DRMSE: Distance Root Mean Squared Error
GSDB: Genome Structure Database

## References

[1] Sati S, Cavalli G (2017). Chromosome conformation capture technologies and their impact in understanding genome function. Chromosoma.

[2] De Wit E, De Laat W (2012). A decade of 3C technologies: insights into nuclear organization. Genes Dev.

[3] Dekker J, Rippe K, Dekker M, Kleckner N (2002). Capturing chromosome conformation. Science.

[4] Han J, Zhang Z, Wang K (2018). 3C and 3C-based techniques: the powerful tools for spatial genome organization deciphering. Mol Cytogenet.

[5] Simonis M, Klous P, Splinter E, Moshkin Y, Willemsen R, De Wit E, Van Steensel B, De Laat W (2006). Nuclear organization of active and inactive chromatin domains uncovered by chromosome conformation capture–on-chip (4C). Nat Genet.

[6] Dostie J, Richmond TA, Arnaout RA, Selzer RR, Lee WL, Honan TA, Rubio ED, Krumm A, Lamb J, Nusbaum C, Green RD (2006). Chromosome Conformation Capture Carbon Copy (5C): a massively parallel solution for mapping interactions between genomic elements. Genome Res.

[7] Lieberman-Aiden E, Van Berkum NL, Williams L, Imakaev M, Ragoczy T, Telling A, Amit I, Lajoie BR, Sabo PJ, Dorschner MO, Sandstrom R (2009). Comprehensive mapping of long-range interactions reveals folding principles of the human genome. Science.

[8] Kalhor R, Tjong H, Jayathilaka N, Alber F, Chen L (2012). Genome architectures revealed by tethered chromosome conformation capture and population-based modeling. Nat Biotechnol.

[9] Li G, Fullwood MJ, Xu H, Mulawadi FH, Velkov S, Vega V, Ariyaratne PN, Mohamed YB, Ooi HS, Tennakoon C, Wei CL (2010). ChIA-PET tool for comprehensive chromatin interaction analysis with paired-end tag sequencing. Genome Biol.

[10] Oluwadare O, Highsmith M, Cheng J (2019). An overview of methods for reconstructing 3-D chromosome and genome structures from Hi-C data. Biological Procedures Online.

[11] Pal K, Forcato M, Ferrari F (2019). Hi-C analysis: from data generation to integration. Biophys Rev.

[12] MacKay K, Kusalik A (2020). Computational methods for predicting 3D genomic organization from high-resolution chromosome conformation capture data. Brief Funct Genomics.

[13] Cournac A, Marie-Nelly H, Marbouty M, Koszul R, Mozziconacci J (2012). Normalization of a chromosomal contact map. BMC Genomics.

[14] Servant N, Varoquaux N, Heard E, Barillot E, Vert JP (2018). Effective normalization for copy number variation in Hi-C data. BMC Bioinformatics.

[15] Imakaev M, Fudenberg G, McCord RP, Naumova N, Goloborodko A, Lajoie BR, Dekker J, Mirny LA (2012). Iterative correction of Hi-C data reveals hallmarks of chromosome organization. Nat Methods.

[16] Knight PA, Ruiz D (2013). A fast algorithm for matrix balancing. IMA J Numer Anal.

[17] Yaffe E, Tanay A (2011). Probabilistic modeling of Hi-C contact maps eliminates systematic biases to characterize global chromosomal architecture. Nat Genet.

[18] Hu M, Deng K, Selvaraj S, Qin Z, Ren B, Liu JS (2012). HiCNorm: removing biases in Hi-C data via Poisson regression. Bioinformatics.

[19] Lyu H, Liu E, Wu Z (2020). Comparison of normalization methods for Hi-C data. Biotechniques.

[20] Trieu T, Oluwadare O, Wopata J, Cheng J (2019). GenomeFlow: a comprehensive graphical tool for modeling and analyzing 3D genome structure. Bioinformatics.

[21] Castellano G, Le Dily F, Hermoso Pulido A, Beato M, Roma G. Hi-Cpipe: a pipeline for highthroughput chromosome capture. bioRxiv. 2015. 10.1101/020636.

[22] Durand NC, Shamim MS, Machol I, Rao SS, Huntley MH, Lander ES, Aiden EL (2016). Juicer provides a one-click system for analyzing loop-resolution Hi-C experiments. Cell Syst.

[23] Servant N, Varoquaux N, Lajoie BR, Viara E, Chen CJ, Vert JP, Heard E, Dekker J, Barillot E (2015). HiC-Pro: an optimized and flexible pipeline for Hi-C data processing. Genome Biol.

[24] Wingett S, Ewels P, Furlan-Magaril M, Nagano T, Schoenfelder S, Fraser P, Andrews S. HiCUP: pipeline for mapping and processing Hi-C data. F1000Res. 2015;4:1310. 10.12688/f1000research.7334.1. 10.12688/f1000research.7334.1 PMC4706059 26835000

[25] Zhang Z, Li G, Toh KC, Sung WK (2013). Inference of spatial organizations of chromosomes using semi-definite embedding approach and Hi-C data. Annual international conference on research in computational molecular biology.

[26] Peng C, Fu LY, Dong PF, Deng ZL, Li JX, Wang XT, Zhang HY (2013). The sequencing bias relaxed characteristics of Hi-C derived data and implications for chromatin 3D modeling. Nucleic Acids Res.

[27] Adhikari B, Trieu T, Cheng J (2016). Chromosome3D: reconstructing three-dimensional chromosomal structures from Hi-C interaction frequency data using distance geometry simulated annealing. BMC Genomics.

[28] Oluwadare O, Zhang Y, Cheng J (2018). A maximum likelihood algorithm for reconstructing 3D structures of human chromosomes from chromosomal contact data. BMC Genomics.

[29] Lesne A, Riposo J, Roger P, Cournac A, Mozziconacci J (2014). 3D genome reconstruction from chromosomal contacts. Nat Methods.

[30] Trieu T, Cheng J (2017). 3D genome structure modeling by Lorentzian objective function. Nucleic Acids Res.

[31] Wang S, Xu J, Zeng J (2015). Inferential modeling of 3D chromatin structure. Nucleic Acids Res.

[32] Zou C, Zhang Y, Ouyang Z (2016). HSA: integrating multi-track Hi-C data for genome-scale reconstruction of 3D chromatin structure. Genome Biol.

[33] Li FZ, Liu ZE, Li XY, Bu LM, Bu HX, Liu H, Zhang CM (2020). Chromatin 3D structure reconstruction with consideration of adjacency relationship among genomic loci. BMC Bioinformatics.

[34] Trieu T, Cheng J (2016). MOGEN: a tool for reconstructing 3D models of genomes from chromosomal conformation capturing data. Bioinformatics.

[35] Kalhor R, Tjong H, Jayathilaka N, Alber F, Chen L (2012). Solid-phase chromosome conformation capture for structural characterization of genome architectures. Nat Biotechnol.

[36] Nowotny J, Ahmed S, Xu L, Oluwadare O, Chen H, Hensley N, Trieu T, Cao R, Cheng J. Iterative reconstruction of three-dimensional models of human chromosomes from chromosomal contact data. BMC Bioinformatics. 2015;16(1):1–9. 10.1186/s12859-015-0772-0 PMC4619219 26493399

[37] Paulsen J, Sekelja M, Oldenburg AR, Barateau A, Briand N, Delbarre E, Shah A, Sørensen AL, Vigouroux C, Buendia B, Collas P (2017). Chrom3D: three-dimensional genome modeling from Hi-C and nuclear lamin-genome contacts. Genome Biol.

[38] Zhu G, Deng W, Hu H, Ma R, Zhang S, Yang J, Peng J, Kaplan T, Zeng J (2018). Reconstructing spatial organizations of chromosomes through manifold learning. Nucleic Acids Res.

[39] Rousseau M, Fraser J, Ferraiuolo MA, Dostie J, Blanchette M (2011). Three-dimensional modeling of chromatin structure from interaction frequency data using Markov chain Monte Carlo sampling. BMC Bioinformatics.

[40] Varoquaux N, Ay F, Noble WS, Vert JP (2014). A statistical approach for inferring the 3D structure of the genome. Bioinformatics.

[41] Kennedy J, Eberhart RC. Particle swarm optimization. In: Proceedings of the 1995 IEEE International Conference on Neural Networks, vol. 4. Piscat away: IEEE Service Center; 1995. p. 1942–1948.

[42] Garcia-Gonzalo E, Fernandez-Martinez JL (2012). A brief historical review of particle swarm optimization (PSO). J Bioinformatics Intelligent Control.

[43] Li MW, Hong WC, Kang HG (2013). Urban traffic flow forecasting using Gauss–SVR with cat mapping, cloud model and PSO hybrid algorithm. Neurocomputing.

[44] Wang J, Hong X, Ren RR, Li TH. A real-time intrusion detection system based on PSO-SVM. In: Proceedings The 2009 International Workshop on Information Security and Application (IWISA 2009), Academy Publisher; 2009. p. 319.

[45] Mohamed MA, Eltamaly AM, Alolah AI (2016). PSO-based smart grid application for sizing and optimization of hybrid renewable energy systems. PLoS One.

[46] Zhang Y, Wang S, Ji G. A comprehensive survey on particle swarm optimization algorithm and its applications. Mathematical problems in engineering. 2015;2015.

[47] Mansour N, Kanj F, Khachfe H (2012). Particle swarm optimization approach for protein structure prediction in the 3D HP model. Interdiscip Sci.

[48] Mohapatra R, Saha S, Dhavala SS. Adaswarm: A novel pso optimization method for the mathematical equivalence of error gradients. arXiv preprint arXiv:2006.09875. 2020.

[49] Bonyadi MR, Michalewicz Z (2017). Particle swarm optimization for single objective continuous space problems: a review. Evol Comput.

[50] Wang G, Guo J, Chen Y, Li Y, Xu Q (2019). A PSO and BFO-based learning strategy applied to faster R-CNN for object detection in autonomous driving. IEEE Access.

[51] Tu C, Chuang L, Chang J, Yang C. Feature Selection using PSO-SVM. IAENG Int J Comput Sci. 2007;33(1):1-6.

[52] Zhang Y, Skolnick J (2004). Scoring function for automated assessment of protein structure template quality. Proteins.

[53] Xu J, Zhang Y (2010). How significant is a protein structure similarity with TM-score= 0.5?. Bioinformatics.

[54] Das K, Jiang J, abd Rao JNK (2004). Mean squared error of empirical predictor. Ann Statist.

[55] Huber PJ. A robust version of the probability ratio test. Ann Math Stat. 1965;36:1753–58.

[56] Duan Z, Andronescu M, Schutz K, McIlwain S, Kim YJ, Lee C, Shendure J, Fields S, Blau CA, Noble WS (2010). A three-dimensional model of the yeast genome. Nature.

[57] Rao SS, Huntley MH, Durand NC, Stamenova EK, Bochkov ID, Robinson JT, Sanborn AL, Machol I, Omer AD, Lander ES, Aiden EL (2014). A 3D map of the human genome at kilobase resolution reveals principles of chromatin looping. Cell.

[58] Oluwadare O, Highsmith M, Turner D, Lieberman-Aiden E, Cheng J (2020). GSDB: a database of 3D chromosome and genome structures reconstructed from Hi-C data. BMC Mol Cell Biol.

[59] Wilke DN. Analysis of the particle swarm optimization algorithm (Doctoral dissertation, University of Pretoria).

[60] Miranda LJ (2018). PySwarms: a research toolkit for Particle Swarm Optimization in Python. J Open Source Software.

[61] Eberhart R, Kennedy J. A new optimizer using particle swarm theory. In: Proceedings of the 6th International Symposium on Micro Machine and Human Science. Nagoya; 1995. pp. 39–43.

[62] Lane J, Engelbrecht A, Gain J (2008). Particle swarm optimization with spatially meaningful neighbours, IEEE Swarm Intelligence Symposium. Sept.

[63] Ni Q, Deng J. A new logistic dynamic particle swarm optimization algorithm based on random topology. Sci World J. 2013;2013:1-8. 10.1155/2013/409167 PMC3683496 23818820

