# Supplementary material for: ParticleChromo3D: a Particle Swarm Optimization algorithm for chromosome 3D structure prediction from Hi-C data
Source: BioData Min. 2022 Sep 21;15:19. doi: 10.1186/s13040-022-00305-x (PMC9494900; doi:10.1186/s13040-022-00305-x)
Supplement: Supplementary file 1 — Additional file 1. [file 13040_2022_305_MOESM1_ESM.docx]

**Supplementary Document for**

**ParticleChromo3D: A Particle Swarm Optimization Algorithm for Chromosome 3D Structure Prediction from Hi-C Data**

David Vadnais^1^, Michael Middleton^1^, and Oluwatosin Oluwadare^1^*

^1^Department of Computer Science, University of Colorado, Colorado Springs, CO, USA.

* Corresponding author

Email addresses:

DV: [dvadnais@uccs.edu](mailto:dvadnais@uccs.edu)

MM: mmiddlet@uccs.edu

OO: [ooluwada@uccs.edu](mailto:ooluwada@uccs.edu)

## Parameters Estimation

### Conversion Factor Test ($\boldsymbol{\alpha}$)


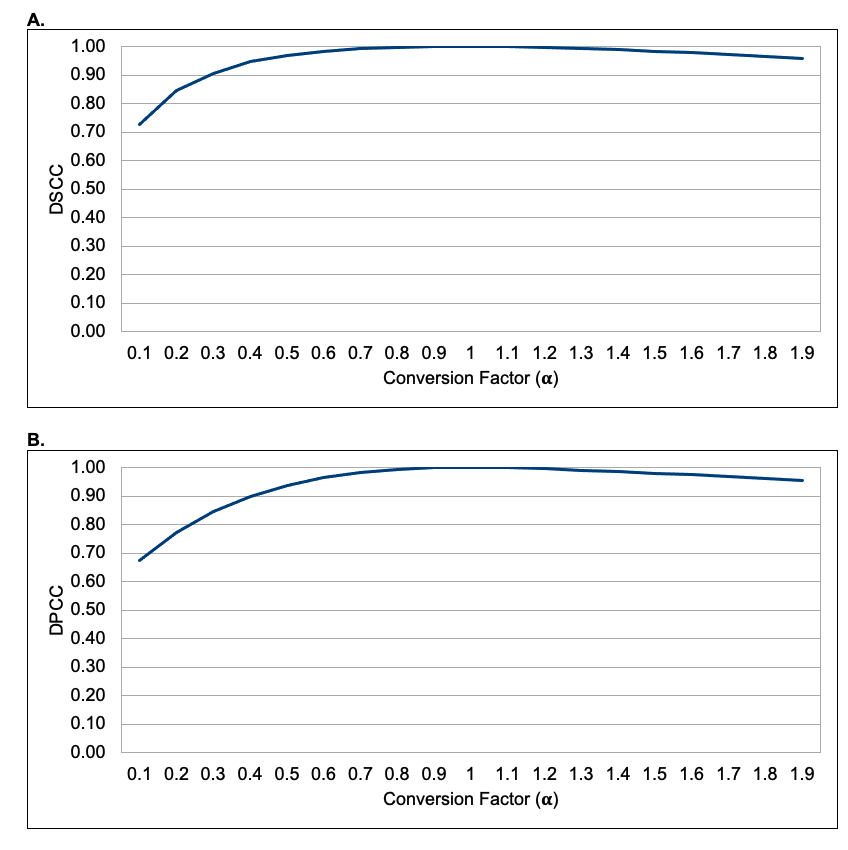


## Figure S1 ­– A plot of the evaluation metric versus the conversion factors.

(A) A plot of DSCC vs. Conversion factor. (B) A plot of DPCC vs. Conversion factor.

Here, we show the performance of ParticleChromo3D on the DSCC and DPCC metric for the simulated dataset at $\alpha$ value in the range 0.1 to 1.9. The result shows the best result is recorded at $\alpha=1$. The DSCC and DPCC metric values were obtained by comparing the ParticleChromo3D algorithm's output structure at each $\alpha$ value with the true structure. In Figure S1A and S1B, the Y-axis denotes the DSCC and DPCC scores, respectively, in the range [-1,1], and the X-axis denotes the conversion factor values. A higher DSCC and DPCC value is better.


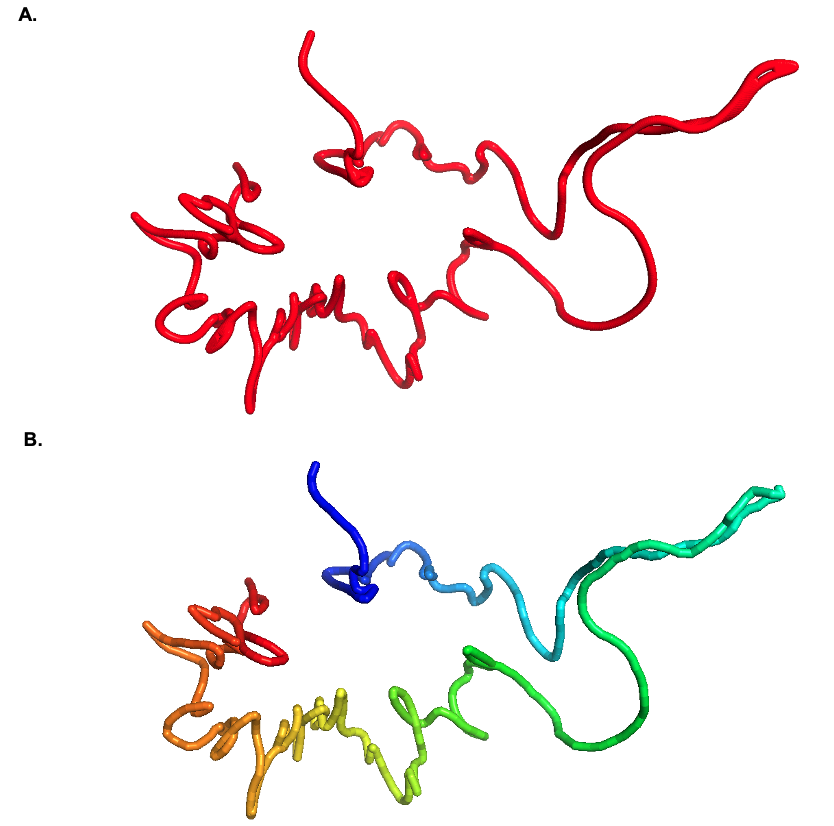


**Figure S2 ­– A comparison of the simulated data true structure and reconstructed structure by ParticleChromo3D.**

(A)True structure from Duan et al. [54] (B) Reconstructed structures for the simulated data using ParticleChromo3D.

### Swarm Size


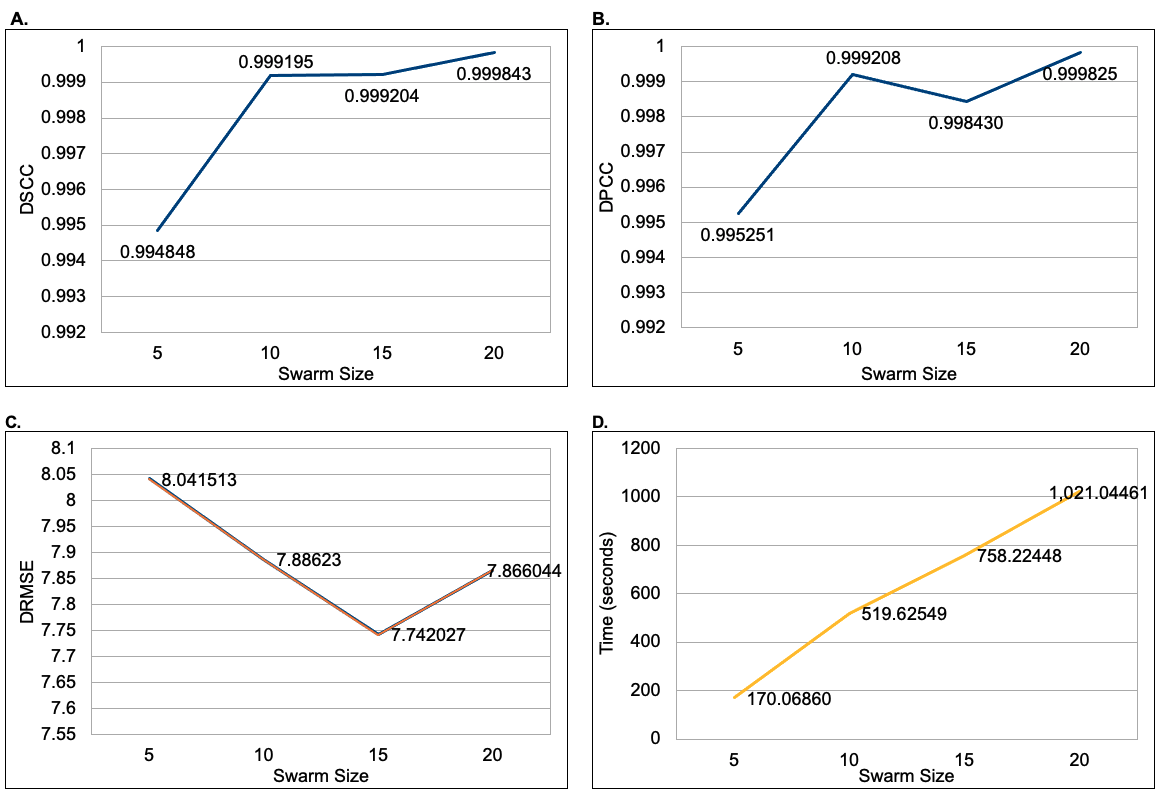


**Figure S3 – A plot of the evaluation metric versus the Swarm Size parameter.**

(A) A plot of the DSCC vs. the Swarm Size. (B) A plot of DPCC vs. the Swarm Size. (C) A plot of DRMSE vs. the Swarm Size. (D) A plot of the runtime, in seconds, vs. the Swarm Size. The DSCC, DPCC, and DRMSE values were obtained by comparing the ParticleChromo3D algorithm's output structure with the simulated data true structure. In Figure S3A and Figure S3B, the Y-axis denotes the DSCC and DPCC score in the range [-1,1], and the X-axis denotes the Swarm Sizes values considered. A higher DSCC and DPCC value is better. In Figure S3C, the Y-axis denotes the DRMSE score, and the X-axis denotes the Swarm Size values. A lower DRMSE value is better. In Figure S3D Y-axis denotes the running time in seconds, and the X-axis denotes the Swarm Size values.


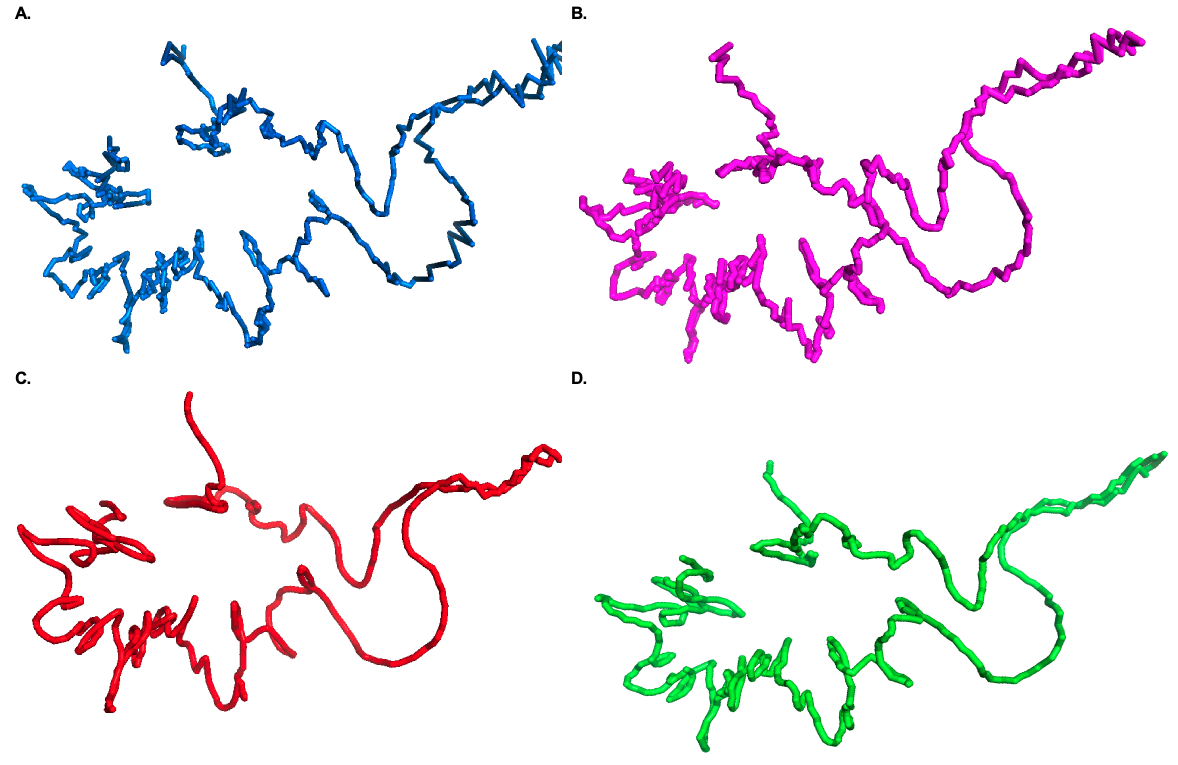


**Figure S4 ­– Structures generated by ParticleChromo3D at different swarm size values.**

Here, we show the structure generated at Swarm size = (A) 5 (Represented with blue color), (B) 10 (Represented with magenta color), (C) 15 (Represented with red color), and (D) 20 (Represented with green color). As shown, the structure generated at swarm size 5 is not smooth; it has a couple of rough edges (Figure S4A). This correlates to the DSCC, DPCC, and DRMSE recorded at this swarm size as it is the lowest at swarm size 5. Next, at Swarm size 10(Figure S4B), we observe a smoother representation but with some rough edges. The result shows that the results were similar at swarm sizes 15 and 20(Figure S4C, Figure S4D).

### Threshold: Optimal parameter to determine structure stability
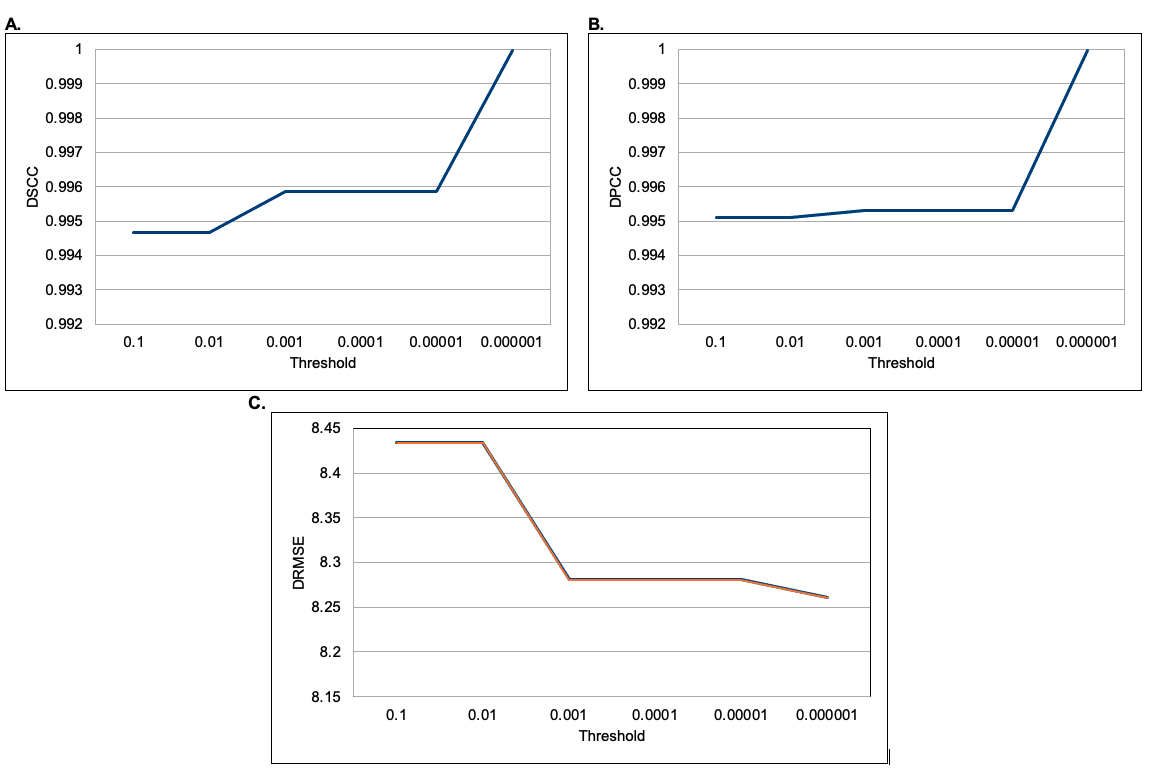


**Figure S5 ­– A plot of the evaluation metric versus the Threshold parameter.**

(A) A plot of the DSCC versus different threshold levels. (B) A plot of DPCC versus the different threshold levels. (C) A plot of DRMSE versus different threshold levels. The results show the performance of our algorithm at threshold values 0.1, 0.01, 0.001, 0.0001, 0.00001, 0.000001. The DSCC, DPCC, and DRMSE values reported were obtained by comparing the ParticleChromo3D algorithm’s output structure to the simulated dataset's true structure. In Figure S5A and S5B, the Y-axis denotes the DSCC and DPCC scores in the range [-1,1], and the X-axis denotes the Threshold values. A higher DSCC and DPCC value is better. In Figure S5C, Y-axis denotes the DRMSE score, and the X-axis denotes the Threshold values. A lower DRMSE value is better.


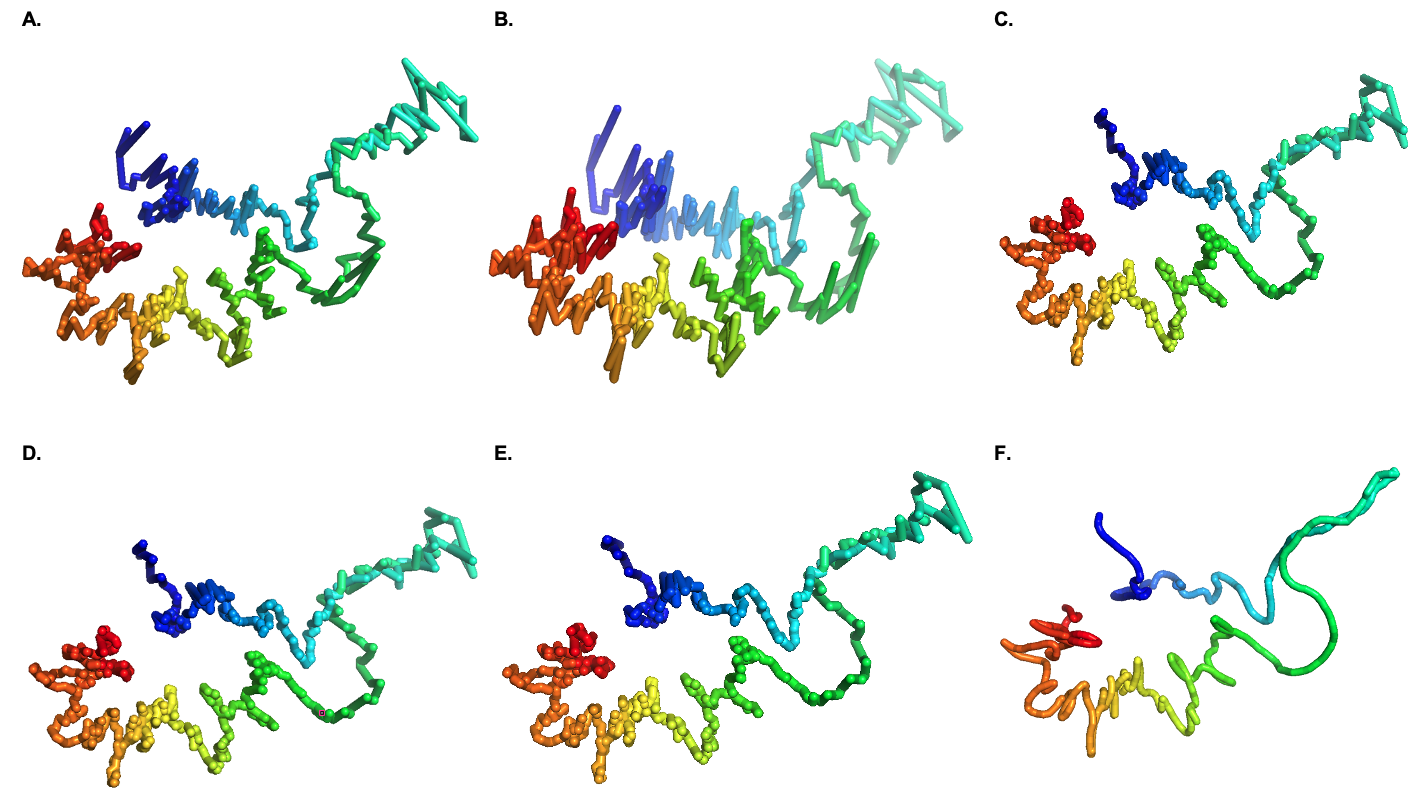


**Figure S6 ­– Structures at a threshold of 0.1, 0.01, 0.001, 0.0001, 0.00001, 0.000001 respectively.**

(A) Represents the structure produced using a threshold of 0.1. (B) Represents the structure produced using a threshold of 0.01. (C) Represents the structure produced using a threshold of 0.001. (D) Represents the structure produced using a threshold of 0.0001. (E) Represents the structure produced using a threshold of 0.00001. (F) Represents the structure produced using a threshold of 0.000001. The results showed that the threshold value of 0.000001, Figure S6F, produced the best result.

### Confidence Coefficient ($\boldsymbol{c}_{\boldsymbol{1}}$ and $\boldsymbol{c}_{\boldsymbol{2}}$)

###
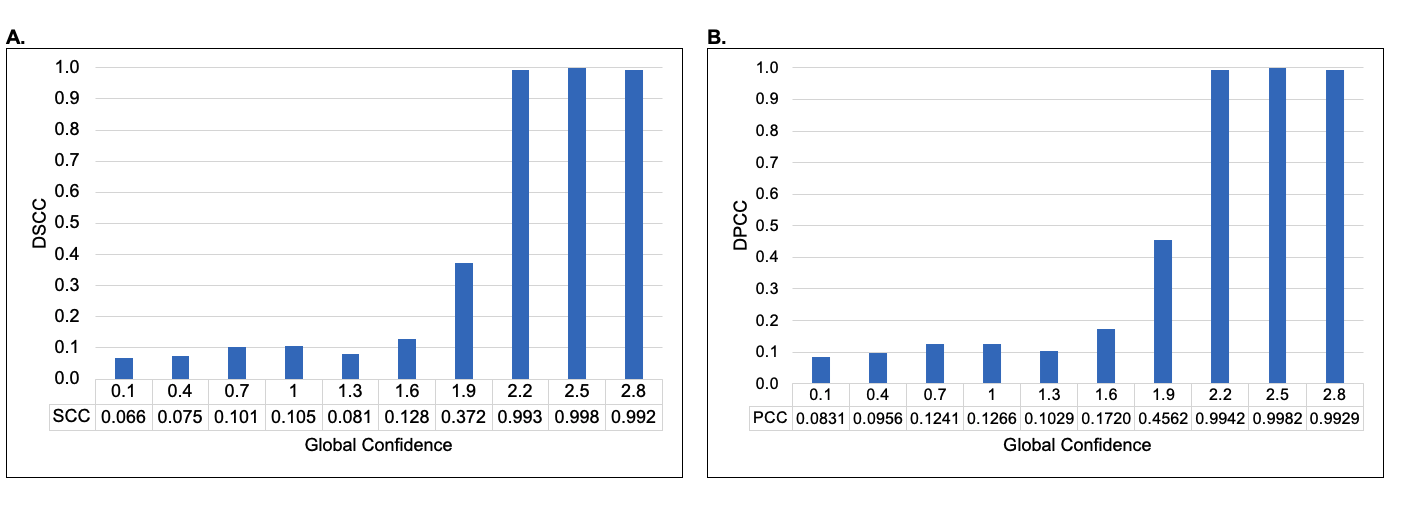


**Figure S7 ­– Confidence Coefficient Test.**

(A) A plot of the DSCC by Global Confidence at Local Confidence 0.3. (B) A plot of DPCC by Global Confidence at Local Confidence 0.3. The plot of the local confidence value local confidence coefficient ($c_{1})=0.3$ against the varying level of global confidence coefficient ($c_{2}$) values from 0.1 to 2.8. The results show that the best result was obtained at $c_{2}$ = 2.5. The DSCC and DPCC values reported were obtained by comparing the ParticleChromo3D algorithm’s output structure with the simulated dataset’s true structure. In Figure S7A and S7B, the Y-axis denotes the DSCC and DPCC scores in the range [-1,1], the X-axis denotes the global confidence values, and the colored plot denotes the local confidence values. A higher DSCC and DPCC value is better.


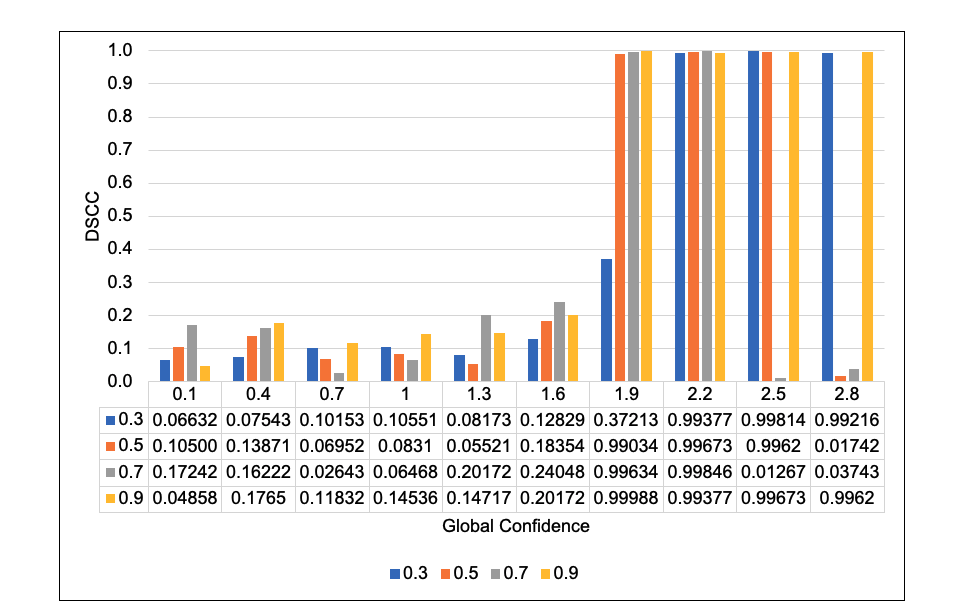


**Figure S8 ­– A combined plot of different local confidences versus global confidences.**

The result's combined plot was obtained by comparing the ParticleChromo3D algorithm’s output structure with the simulated dataset’s true structure for local confidence values of 0.3 to 0.9 and global confidence values of 0.1 to 2.8. This plot shows the DSCC accuracy of the structures generated. The Y-axis denotes the DSCC score in the range [-1,1], the X-axis denotes the global confidence values, and the colored plot denotes the local confidence values. A higher DSCC value is better.


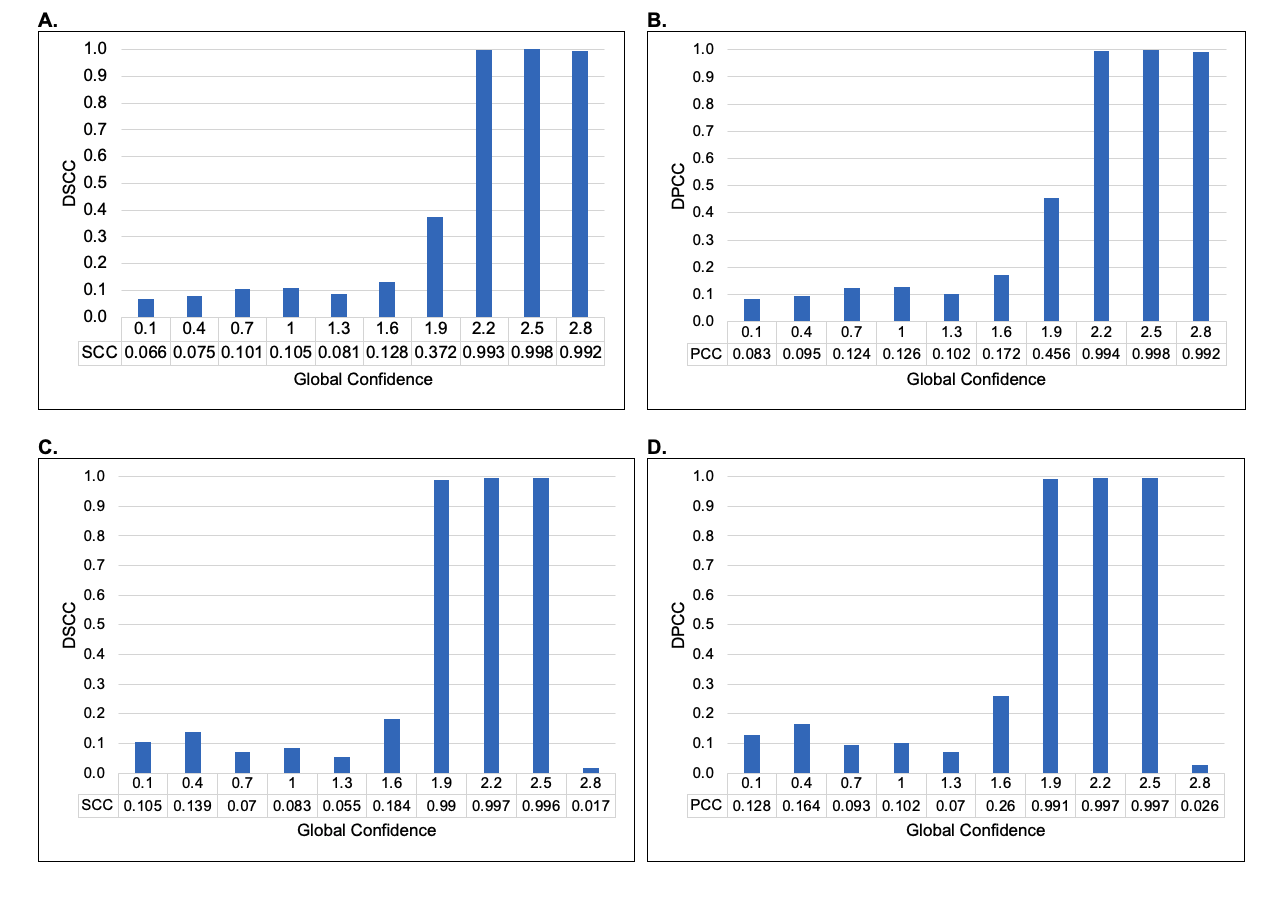


**Figure S9 – A Plot of local confidence values 0.3 and 0.5 versus global confidence.**

(A) DSCC by Global Confidence at Local Confidence 0.3 (B) DPCC by Global Confidence at Local Confidence 0.3. (C) DSCC by Global Confidence at Local Confidence 0.5 (D) DPCC by Global Confidence at Local Confidence 0.5. Each of the plots shows the DSCC and DPCC results obtained by comparing the ParticleChromo3D algorithm's output structure with the simulated dataset's true structure for local confidence values 0.3 to 0.5 and global confidence values 0.1 to 2.8. The Y-axis denotes the DSCC or DPCC scores, respectively, as a label in the title, in the range [-1,1], the X-axis denotes the global confidence values, and the colored plot denotes the local confidence values. A higher DSCC and DPCC value is better.


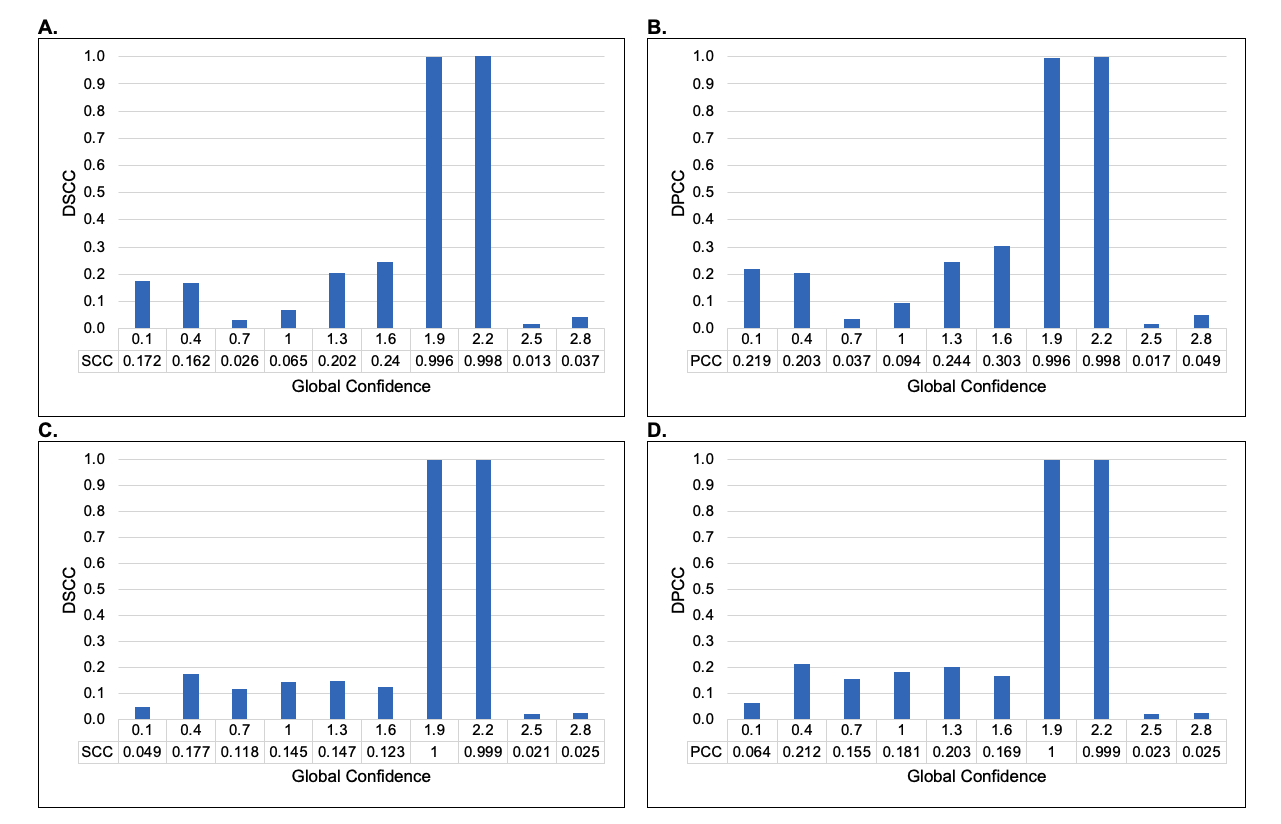


**Figure S10 – A Plot of local confidence values 0.7 and 0.9 versus global confidence.**

(A) DSCC by Global Confidence at Local Confidence 0.7. (B) DPCC by Global Confidence at Local Confidence 0.7. (C) DSCC by Global Confidence at Local Confidence 0.9. (D) DPCC by Global Confidence at Local Confidence 0.9. Each of the plots shows the DSCC and DPCC results obtained by comparing the ParticleChromo3D algorithm’s output structure with the simulated dataset’s true structure for local confidence values 0.7 to 0.9 and global confidence values 0.1 to 2.8. The Y-axis denotes the DSCC or DPCC scores, respectively, as a label in the title, in the range [-1,1], the X-axis denotes the global confidence values, and the colored plot denotes the local confidence values. A higher DSCC and DPCC value is better.

## Topologies


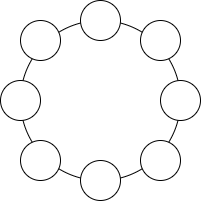


**Figure S11 ­– Ring topology diagram**

We present an illustration of the layout of a ring topology. This topology is used by Local best optimization.


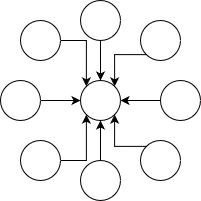


**Figure S12 ­– Star topology diagram**

We present an illustration of the layout of a star topology.


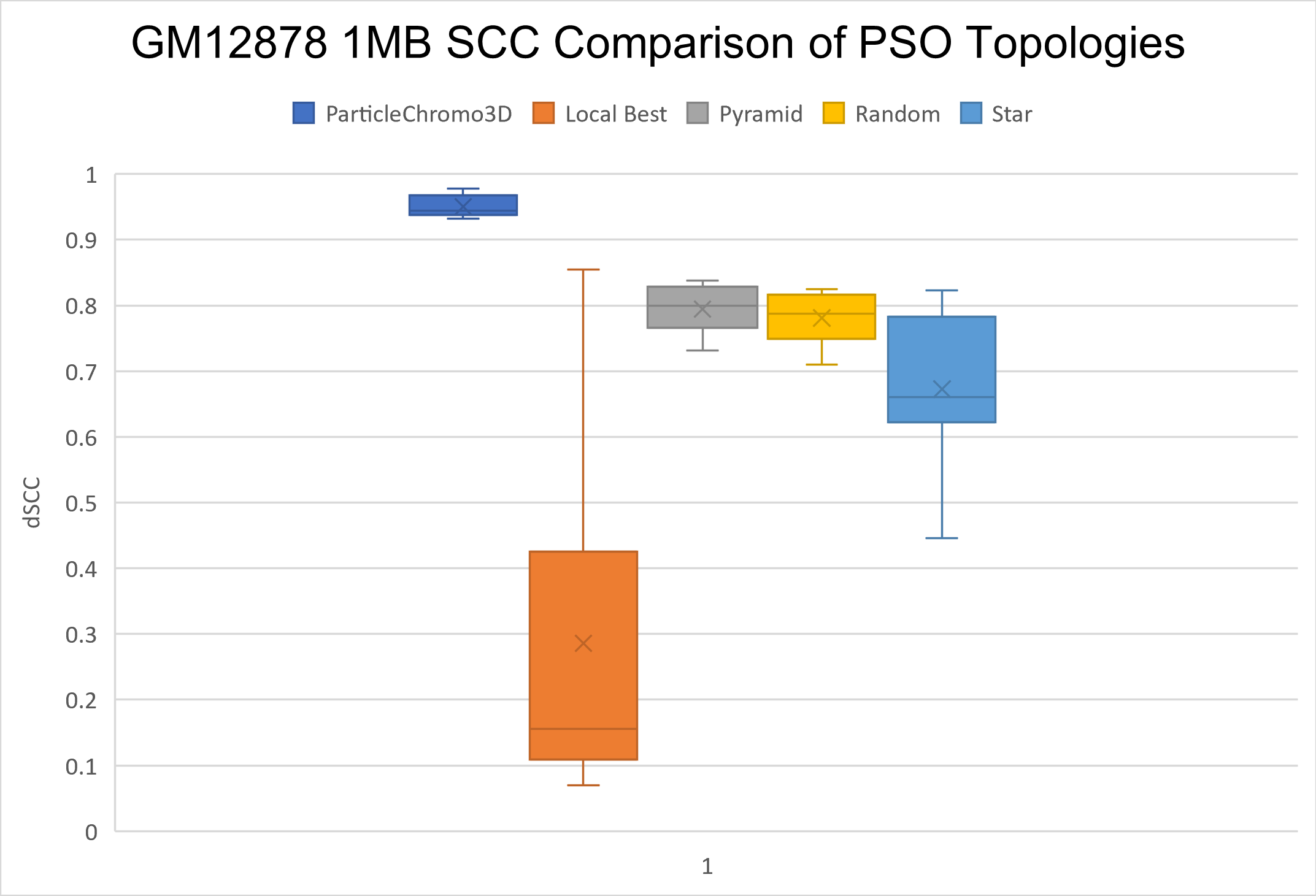


**Figure S13 – ParticleChromo3D DSCC performance Compared to other topologies 1MB GM12878 cell Hi-C data.**

Comparing the performance by ParticleChromo3D on the 1MB GM12878 cell Hi-C data compared to open-source optimizer topologies. We only analyzed chromosomes 16-23. The Y-axis denotes the DSCC metric score in the range [-1,1], and X-axis denotes the topology. A higher DSCC value is better.
